# Multilevel Interventions Demonstrate Mixed Effectiveness for Improving Blood Pressure Outcomes: A Rapid Review

**DOI:** 10.3390/healthcare13121397

**Published:** 2025-06-11

**Authors:** Briana N. Sprague, Anna K. Forster

**Affiliations:** 1 Division of General Internal Medicine and Geriatrics, Indiana University School of Medicine, Indianapolis, IN 46202, USA; 2 School of Nursing, Indiana University Indianapolis, Indianapolis, IN 46202, USA; akforste@iu.edu

**Keywords:** blood pressure, multilevel intervention, cardiovascular health, adults, randomized controlled trial, health promotion

## Abstract

Objective: What types of multilevel interventions exist to improve blood pressure among community-dwelling adults aged 18+ in the United States? What is the treatment efficacy? Data Source: Peer-reviewed articles from Cochrane Library, EMBASE, PsycINFO, and PubMed. The search strategy was pre-registered on Open Science Framework. Study Inclusion and Exclusion Criteria: Inclusion criteria were community-dwelling adults in the United States aged 18 or older; interventions involving at least two levels; at least one blood pressure outcome measured; and published in a peer-reviewed journal. Data Extraction: Intervention activities, blood pressure outcomes, and moderation/subgroup analyses, when available, were extracted. Data Synthesis: Qualitative synthesis and summary statistics. Results: Ninety-five papers covering 89 RCTs were included. Multilevel interventions involving the individual and healthcare team (without health policies = 49 studies; with health policies = 15 studies) tended to show the most consistent saltatory effects on blood pressure (systolic: 46% of studies showed statistical improvement; diastolic: 47% of studies showed statistical improvement). Interventions involving families or communities outside of healthcare settings were promising but were less frequently reported (19% of studies). Conclusions: There was mixed evidence that multilevel interventions targeting cardiovascular health improved blood pressure among U.S.-based adults. Future research should continue evaluating interventions that improve the individual as well as the environments in which individuals work and play, especially those levels outside of traditional healthcare settings.

## 1. Introduction

Despite population-level improvements in prevalence and control of hypertension among adults, disparities among subpopulations including non-Hispanic Black adults persist [1]. Current first-line treatments for elevated blood pressure (BP) and hypertension primarily involve individual lifestyle modifications such as diet and nutritional changes, physical activity, or weight loss [2]. While these treatments improve BP, there is limited evidence they reduce disparities or address social determinants of adoption/adherence of healthy lifestyle behaviors [3,4]. Multilevel interventions, or those affecting multiple levels of influence such as an individual and their healthcare provider [5], may outperform solely individual-based interventions for BP as they better account for one’s social or physical environment [6]. A multilevel approach may also reduce health disparities BP by intervening on larger structural factors (e.g., health policies). The last systematic review on blood pressure (BP) control interventions was published in 2018 and primarily focused on pharmacologic or team-based healthcare interventions. However, since then, there have been growing calls from organizations like the American Heart Association (AHA) to address upstream factors influencing BP, including stress and social determinants of health [7]. These upstream factors include social determinants such as housing insecurity, neighborhood conditions, food access, economic instability, chronic stress, and systemic racism [8,9,10,11]. While these are widely recognized as root causes of hypertension disparities, most interventions continue to emphasize individual behavior change. Our review extends the prior literature by including multilevel interventions across diverse domains—healthcare teams, families, communities, and health policies—which reflect the evolving landscape of BP management strategies. By incorporating studies before and after 2018, we comprehensively assess intervention effectiveness while identifying persistent gaps in addressing disparities in BP control, particularly among minoritized groups. In response to recent calls from the AHA to rigorously examine multilevel interventions with the greatest potential for reducing cardiovascular health disparities [12], we conducted a rapid review to answer the following:

(1) What types of multilevel interventions exist to improve BP among community-dwelling adults aged 18 years+ in the United States (U.S.)?
(2) What is the treatment efficacy on BP?

Where possible, we also described which study characteristics moderated intervention efficacy.

## 2. Methods

Our review followed Grant and Booth’s [13] approach to conducting a rapid review and was conducted in line with the reporting guidelines of the Preferred Reporting Items for Systematic Reviews and Meta-Analyses (PRISMA) statement (Appendix A.2) [14]. Methodological quality was assessed using the Cochrane Risk of Bias (RoB) Tool version 2.0 for RCTs [15]. This tool evaluates five domains: (1) the randomization process, (2) deviations from intended interventions, (3) missing outcome data, (4) measurement of the outcome, and (5) selection of the reported result. Each domain was rated as “Low risk”, “Some concerns”, or “High risk”, and an overall risk of bias judgment was determined according to Cochrane guidance, typically reflecting the highest risk rating across domains. Study quality assessment was used to interpret results synthesized in this review and to guide the development of conclusions and future recommendations. Full item-level RoB scores, with reviewer justifications for each domain, are publicly available via the study’s Open Science Framework page: https://osf.io/9nhef.

Prior to executing database searches, we preregistered our protocol on Open Science Framework (OSF). An anonymized OSF page is available at: https://osf.io/hzsr6/?view_only=30b08c6976774136aefd04f70f14f8f0 (accessed on 1 February 2024).

## 3. Data Sources

An exhaustive search of 4 databases was conducted. Databases included were PubMed, EMBASE, Cochrane Library (including Cochrane Reviews), and PsycINFO. The search strategy was piloted and refined following the advice of a medical librarian. Searches were conducted between 24 January and 2 February 2024. Search terms for each database are provided in Appendix A.1. Search terms were selected to reflect the following: (1) BP, hypertension, or CVD health assessed by the AHA’s Simple 7 or its updated version, the Essential 8 [16]; (2) multilevel interventions; and (3) adults aged 18 or older living in the U.S. Initial search results were downloaded into the Covidence software (https://support.covidence.org/help/how-can-i-cite-covidence, accessed on 1 February 2024) program to remove duplicates (Covidence systematic review software, Veritas Health Innovation, Melbourne, Australia. Available at www.covidence.org, accessed on 1 February 2024).

## 4. Inclusion and Exclusion Criteria

Studies. Studies were selected based on the following major inclusion criteria: (1) participants were community-dwelling adults aged 18 years or older based in the U.S.; (2) included a multilevel intervention evaluated in an RCT with an appropriate control group; (3) included at least one BP-related outcome; and was (4) published any time in a peer-reviewed journal. As this was a rapid rather than systematic review, interventions published in grey literature or in a non-English language were excluded.

In an intervention context, multilevel interventions are those that target at least two domains [5,6]. Informed by ecological frameworks that inform behavioral interventions, we categorized intervention activities into the following domains: (a) individuals, (b) family or other social supports; (c) healthcare teams (e.g., physician, pharmacist, nurse); (d) community environment (e.g., specific community stakeholders or physical environments themselves); or (e) health policies (e.g., decision support aids) [17,18]. In the literature, community health workers or promotores were frequently included as part of the intervention. We classified them as either belonging to the healthcare team or community environment, depending on which setting the community health workers/promotores conducted their intervention activities. See Table 1 for the major inclusion criteria and sample characteristics of the studies that were included in the current review. 

Studies were excluded for various reasons, even if they initially appeared to meet the inclusion criteria. For example, studies that included interventions targeting only one domain (e.g., individual-level interventions) were excluded, as they did not meet the multilevel intervention criterion. Additionally, some studies focused on populations outside of the U.S. or included individuals in institutional settings, which led to their exclusion. We also excluded studies where the intervention did not meet the definition of multilevel, such as those that did not integrate multiple intervention domains (e.g., individual and healthcare team or individual and community).

Participants. Interventions were eligible if their participants were community-dwelling adults in the U.S. aged 18 years+. By community-dwelling adults, we refer to those not living in institutionalized settings such as skilled nursing facilities, nursing homes, etc. This population represents a crucial focus for public health interventions, as they are likely to benefit from community-based resources and preventive strategies. Interventions targeting community-dwelling adults can also address upstream factors such as social support and stress that play key roles in blood pressure control, especially among underserved populations. Participants did not need a diagnosed BP-related health condition (e.g., elevated BP or a history of hypertension diagnosis from electronic medical records) for inclusion in this review. We included studies where participants did not necessarily have diagnosed BP-related conditions to capture interventions aimed at primary prevention. Many social and behavioral determinants, such as chronic stress and limited social support, contribute to elevated BP long before clinical diagnosis; including these studies allows for a broader understanding of intervention effectiveness across the continuum of BP risk, addressing both prevention and management. If studies included individuals under 18 (e.g., parent–child intervention), the study was eligible for inclusion, and only adult outcomes were reported.

Primary and secondary outcomes. For each included study, we extracted all reported blood pressure outcomes. This included in-office blood pressure measurements, ambulatory blood pressure monitoring (ABPM), home blood pressure monitoring, clinical diagnosis of hypertension, and composite cardiovascular health scores where blood pressure was a component (such as Life’s Simple 7 or Life’s Essential 8). When studies reported multiple blood pressure outcomes, all eligible outcomes were extracted. Extracted effect measures included raw blood pressure values (reported as mean ± standard deviation when available), proportions of participants classified as hypertensive (based on study-defined criteria), and cardiovascular risk scores where blood pressure was embedded in composite measures (e.g., Life’s Simple 7 or Essential 8). Due to variation in how outcomes were reported across studies, results were summarized narratively and using descriptive statistics.

Other data items. In addition to blood pressure outcomes, we extracted study-level variables including the following: participant characteristics (age, gender, race/ethnicity, and comorbidities); intervention characteristics (delivery setting, duration, intensity, target levels [individual, healthcare team, family, community, policy]); and reported subgroup or moderator analyses, when available. If data were unclear or not reported, assumptions were avoided, and missingness was noted in the extraction table.

## 5. Data Extraction

Searches were conducted, screened, and reviewed for inclusion according to the selection criteria by one author (A1). Data were extracted concurrently with the risk of bias (RoB) assessment by two authors (A1, A2). Rather than independently assessing all articles, the authors divided them for data extraction and RoB assessment and met regularly to adjudicate articles. Extracted data included overall sample size, intervention activities, intervention duration, comparison group(s), key eligibility criteria, and results (Table 2 and Table 3). Study-level RoB item scores are available via the study’s Open Science Framework page (https://osf.io/hzsr6/?view_only=30b08c6976774136aefd04f70f14f8f0, accessed on 1 February 2024). Missing or incomplete data were noted in the data extraction table, and no assumptions were made regarding these missing data. Studies with unclear or missing outcomes were excluded from relevant analyses, and such exclusions are detailed in Appendix A. Formal assessment of reporting bias (e.g., publication bias or selective outcome reporting) was not conducted, consistent with the narrative nature and methodological scope of this rapid review.

## 6. Data Synthesis

Findings from the included studies were synthesized using tables, summary statistics, and narrative summaries. Studies were grouped based on the intervention components: (1) individual and healthcare team; (2) individual and healthcare team and health policy; (3) individual and community environment; (4) individual and family/social support; and (5) combinations of these components, as described in the inclusion and exclusion criteria. This grouping allowed us to compare the effectiveness of different types of multilevel interventions on BP outcomes. Results from the included studies were summarized using tables (Table 2 and Table 3), which detail study characteristics, outcomes, and intervention types. In addition, summary statistics for blood pressure outcomes and moderator analyses were provided, accompanied by narrative synthesis to highlight key findings across intervention types.

As a rapid review, findings were synthesized using a narrative summary approach, which involved qualitative analysis and summary statistics to provide a descriptive overview of the results. Given the heterogeneity of the included studies (in terms of intervention types and outcomes), meta-analysis was not performed. The synthesis aimed to assess intervention effectiveness across different components and moderators, as detailed in Table 2 and Table 3.

We conducted subgroup analyses to examine the effect of baseline blood pressure on intervention effectiveness. These analyses were performed when studies provided data on baseline BP and were consistent with our review’s objectives. Given the narrative nature of this review, no formal statistical tests for heterogeneity were performed.

## 7. Results

**Figure 1:**
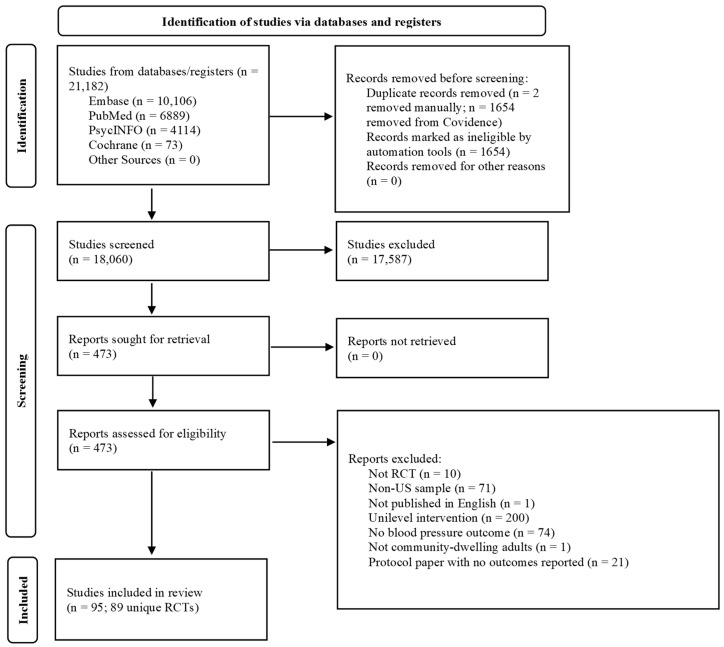
PRISMA study selection flow chart.

The PRISMA flow chart (Figure 1) shows the studies’ selection process. The literature search yielded 21,182 articles, of which 473 full-text articles were screened for eligibility. Ninety-five articles reporting on 89 multilevel interventions were ultimately included. All studies were randomized at either the individual or cluster level. All interventions were conducted in the U.S. with adults aged 18 or older (Table 1).

## 8. Sample Characteristics

The sample size of the included studies ranged from 35 to 10,698 participants distributed across 93 healthcare providers. There was marked heterogeneity across samples by race, gender, and study-specific eligibility criteria (e.g., eligibility depending on diagnosis of hypertension or Type 2 diabetes). Common participant-specific eligibility criteria across studies included the following: (1) the presence or absence of hypertension, CVD disease, type 2 diabetes, obesity, or participant is prescribed medication to treat these health conditions (e.g., antihypertensives); (2) elevated lab measures such BP or HbA_1c_; or (3) demonstrated poor BP control evinced through medication refill audits or insufficient medication intensification. Twenty-five of the 95 studies (26%; 22 RCTs and 2 follow-up manuscripts) included racial or ethnic identity as an inclusion criterion; this included Black (17%; 14 RCTs and 2 follow-up manuscripts) or South Asian (1%; 1 RCT) adults or those with Hispanic/Latinx ethnicity (7%; 7 RCTs). Overall, 6 of the 95 studies (6%; 4 RCTS and 2 follow-up manuscripts) were conducted only among men, and 3 studies (3%; 3 RCTs) were conducted only among women.

For cluster-randomized interventions, the inclusion criteria ranged but tended to include the following: (1) years or hours/week a physician sees patients; (2) a percentage of the site’s caseload or clientele meeting eligibility criteria (e.g., uncontrolled BP); or (3) the site serves a particular population (e.g., predominantly Black churches or barbershops).

## 9. General Characteristics of the Multilevel Interventions

Interventions varied in length from four weeks to five years. The most common intervention duration was 12 months (38 RCTs; 40%). Interventions were most often compared against (enhanced) usual care (67 studies; 70%). Other control group types included delayed intervention/waitlist, psychoeducation (e.g., receive pamphlet with Joint National Committee [JNC]-7 guidelines [114]), or low-intensity versions of the multilevel intervention, which may include individual intervention components in a factorial design or less intensive dose in a nonfactorial design.

Most studies had low RoB (67%; 60 RCTs, 3 follow-up papers, and 1 subgroup analysis), but a sizeable proportion also had some RoB concerns (31%; 29 RCTs and 1 follow-up paper). Only one study had high RoB.

In descending frequency, the types of multilevel interventions targeting BP outcomes were: (1) individual and healthcare team (47 RCTs and 2 follow-up papers); (2) individual and healthcare team and health policies (15 RCTs; no follow-up papers); (3) individual and community environment (9 RCTS; no follow-up papers); (4) individual and family/social support (6 RCTs and 1 follow-up paper); (5) individual and family/social support and healthcare team (4 RCTs; no follow-up papers); (6) healthcare team and health policies (3 RCTs; no follow-up papers); (7) individual and healthcare team and community environment (2 RCTs, 1 follow-up paper, and 1 subgroup analysis); (8) individual and community environment and health policies (2 RCTs; no follow-up papers); (9) individual and health policies (1 RCT; no follow-up papers), and (10) individual and family/social support and healthcare team and community environment (1 RCT, no follow-up papers). Notably, all but three RCTs [103,104,105] incorporated individual level intervention activities. Across the 89 randomized controlled trials included in this review, over half (53%) were published in 2018 or later, reflecting a relatively recent body of evidence. For readability, descriptions of intervention activities and outcomes are separated by intervention type. See Table 2 and Table 3 for additional details of intervention activities and outcomes. For a summary of multilevel interventions showing robust or sustained blood pressure improvements and population-specific effects, see Appendix A.3.

### 9.1. Individual and Healthcare Team (47 RCTs and 2 Follow-Ups)

Blood pressure outcomes. In total, 45 studies (43 RCTs and 2 follow-up papers) examined systolic BP as an outcome measure. Of those, 23 studies (51%; 21 RCTs and 2 follow-up papers) found significantly lower systolic BP among the intervention group.

Thirty-one studies (30 RCTs and 1 follow-up paper) examined diastolic BP as an outcome measure. Of those, 17 (55%; 16 RCTs and 1 follow-up paper) found significantly lower diastolic BP among the intervention group. Six studies (6 RCTs, no follow-ups) examined other BP outcomes reflecting adequate BP control at the individual or clinic level, of which half (3 RCTs; 50%) found significantly better BP outcomes among the intervention group. Including the 2 follow-up papers, 6 RCTs examined post-intervention maintenance effects. Five studies (80%) found that immediate post-intervention benefits were not sustained, and one found long-term but not immediate post-intervention improvement.

Moderators. Three studies examined whether intervention effects were dependent on one’s baseline BP values. In two studies (66%), the intervention was significantly more effective for those with higher baseline BP/less adequate BP control. In the third study, there was a larger, albeit statistically nonsignificant, improvement among participants with poorer baseline BP control. Age, gender, and which team member delivered the intervention were examined as moderators in only one study, so no strong conclusions can be drawn about their effect on intervention effectiveness.

### 9.2. Individual and Healthcare Team and Health Policy (15 RCTs and 0 Follow-Ups)

Blood pressure outcomes. Thirteen studies examined systolic BP as an outcome measure. Of those, 4 studies (31%) found significantly lower systolic BP among the intervention group. Three studies examined whether intervention effects on systolic BP were maintained; intervention effects were sustained in one study (33%). Seven studies examined diastolic BP as an outcome measure. Of those, one study (14%) found significantly lower diastolic BP among the intervention group. Four studies examined other BP outcomes, including medication refill adherence, physician clinical inertia, or percentage of patients with adequate BP control. Three studies (75%) found significantly better BP outcomes among the intervention group.

Moderators. One study examined whether the intervention significantly improved systolic BP when restricting analyses to those with baseline systolic BP ≤ 130. Like the full-sample analyses, there were no significant intervention effects. No other moderators were examined among these interventions.

### 9.3. Individual and Community Environment (9 RCTs and 0 Follow-Ups)

Blood pressure outcomes. All studies examined intervention effects on systolic BP, with significant intervention effects in two studies (22%). Improvements to systolic BP were not present in one study that also reported 12-month outcomes. All but one study examined intervention effects on diastolic BP, with significant intervention effects not sustained at 12 months in one study (12.5%). Two studies examined intervention effects on Life’s Simple 7 total scores, with significant intervention effects present in both.

Moderators. One study examined the moderating effect of age on total Life’s Simple 7 improvement and found significant intervention effects only among those participants under 60 years old.

### 9.4. Individual and Family (6 RCTs and 1 Follow-Up)

Blood pressure outcomes. Six RCTs (and 1 follow-up) examined intervention effects on systolic BP, and no significant intervention effects were found, nor did they occur at follow-up. Four RCTs (and 1 follow-up) assessed diastolic BP, where intervention effects were mixed. In one RCT, children but not parents had significantly improved diastolic BP. A second RCT (and 1 follow-up) demonstrated significantly improved diastolic BP for the care recipient’s partner only. This improvement was sustained only among those who received couples counseling. The remaining two studies did not find significantly improved diastolic BP. Additionally, one study assessed 30-year estimated systolic BP and found marginally significant improvement for the care recipient’s partner.

Moderators. Intervention effect moderators were not examined in these studies.

### 9.5. Individual and Family and Healthcare Team (4 RCTs and 0 Follow-Ups)

Blood pressure outcomes. All studies measured systolic BP, with mixed evidence of improvements among the intervention relative to control group (25% with improvement). Two studies measured diastolic BP and found mixed evidence for significant improvements in the intervention relative to control group (50% with improvement).

Moderators. Intervention effect moderators were not examined in these studies.

### 9.6. Healthcare Team and Health Policy (3 RCTs and 0 Follow-Ups)

Blood pressure outcomes. One study assessed systolic BP and found significant improvements in daytime, nighttime, and 24 h systolic BP in the intervention relative to control group. Two studies assessed the percentage of patients who had controlled BP; one found physician-level incentives, but not practice-level or combined physician-practice incentives resulted in greater BP control that was not sustained after an 18-month washout period. A second study found the intervention relative to control had more participants with controlled 24 h BP at immediate posttest. Lastly, one study assessed the percentage of patients who met target BP levels and found significantly greater BP control among intervention clinics.

Moderators. Intervention effect moderators were not examined in any study.

### 9.7. Individual and Healthcare Team and Community Environment (2 RCTs and 1 Follow-Up and 1 Subgroup Analysis)

Blood pressure outcomes. Both RCTs assessed immediate posttest systolic BP, where there was mixed evidence of the intervention such that there was significant improvement in one and marginal improvement in the other. For the RCT with significant immediate systolic BP changes, improvements were sustained at 12 months posttest. Both RCTs also assessed immediate posttest diastolic BP, where there was similarly mixed evidence of the intervention such that there was significant improvement in one and no improvement in the other. For the RCT with significant immediate diastolic BP changes, improvements were sustained at 12 months posttest.

Moderators. One study examined effects of the barber program among a subgroup of men with a baseline systolic BP ≥ 140 mmHg and found those whose barbers referred them to a hypertension specialist had significantly improved systolic BP compared to controls. There were no significant intervention effects among participants whose barbers referred them to primary care physicians.

### 9.8. Individual and Community Environment and Health Policies (2 RCTs and 0 Follow-Ups)

Blood pressure outcomes. Both studies examined the intervention effect on systolic BP, and neither study demonstrated significant improvement relative to controls. Both studies examined diastolic BP, but there were also no significant intervention effects for either relative to controls.

Moderators. One study restricted analyses to those with dysglycemia and found that the intervention effect on systolic BP was stronger, albeit statistically nonsignificant, compared to the full sample.

### 9.9. Individual and Health Policy (1 RCT and 0 Follow-Ups)

Blood pressure outcomes. Systolic BP improved similarly across intervention groups, but there were no between-group differences.

Moderators. Intervention effect moderators were not examined in this study.

### 9.10. Individual and Family and Healthcare Team and Community (1 RCT and 0 Follow-Ups)

Blood pressure outcomes and moderators. The BP outcomes reported were from when the children were assessed in their mid-30s. Men who received the program had significantly better systolic and diastolic BP compared to controls and were less likely to fall into the Stage I hypertension category. Women did not have significantly different BP values but were marginally less likely to be pre-hypertensive.

## 10. Discussion

Despite ongoing efforts to improve BP within the U.S., rates of uncontrolled BP and hypertension prevalence have increased in recent years [115,116]. Although interventions targeting individual-level CVD risk factors such as physical activity are promising therapeutic approaches, achieving optimal BP control involves a variety of individual, social, environmental, and healthcare system factors. We found overall, there was mixed evidence that multilevel interventions targeting such factors improved BP outcomes among community-dwelling adults in the U.S. Multilevel interventions involving the individual and healthcare team—either with or without health policy intervention components—tended to show the most consistent saltatory effects on BP, but emerging literature suggests multilevel interventions involving the community hold promise for improving BP as well. In addition to immediate posttest effects, only a minority of studies assessed outcomes beyond 12 months, and even fewer examined whether improvements were sustained following the active intervention period. Effects that were reported often diminished over time, supporting existing evidence that ongoing support and maintenance strategies are needed to preserve BP-reducing benefits. This underreporting of long-term and post-intervention outcomes limits our ability to draw conclusions about the durability of multilevel interventions and highlights the need for extended follow-up in future trials [15].

Almost all interventions included activities for the individual/patient to engage in and usually targeted traditional CVD risk factors such as smoking cessation, participating in fitness courses, or receiving BP self-monitoring psychoeducation and equipment. While individual-based lifestyle modification improves CVH, making these changes without comprehensive efforts to address the underlying social conditions that drive high-risk health behaviors is very difficult and may exacerbate existing inequities [117,118].

The multilevel interventions in this review attempt to address upstream factors as they occur within the healthcare setting (e.g., people or policies) or community. The healthcare team was frequently activated and involved collaborative care between members of a healthcare team and occurred within traditional healthcare settings. Teams typically included embedded physicians, pharmacists, and community health workers/promotores. The role of the community health worker was variable across interventions [19]; not only could they help patients navigate the healthcare system, but they also supported navigating patients’ local neighborhood facilities and resources. When healthcare policies were enacted, they typically involved electronic decision support aids, physician-level incentives for controlling their patients’ BP, or other incentive-related policies to promote health behaviors. Although less commonly applied, there were interventions involving people and places outside of a traditional healthcare setting, such as churches and barbershops, that primarily serve racially minoritized populations. Like community health workers, these individuals were typically community members trained on skills like delivering health information, discussing barriers and facilitators to achieving health, and facilitating resource linking/referrals for participants. Interventions involving family were also not commonly applied but show promise for having short-term benefits, as well as the potential to mitigate long-term CVH decline and health disparities across ages [118]. Together, interventions using these approaches provide models for establishing partnerships between traditional healthcare providers and individuals within the communities they serve. Notably, few studies rigorously evaluated policy-level interventions through randomized controlled trials, limiting our understanding of how systemic changes—such as insurance coverage expansion, clinician reimbursement models, or public health infrastructure investments—might interact with individual or healthcare team-level efforts to improve BP control.

These gaps in equity-focused analysis are further underscored by the limited exploration of treatment moderators across trials. While many interventions were conducted in racially minoritized populations, few explicitly tested whether effects varied by race, ethnicity, or socioeconomic status. A small number of studies, such as Victor et al. [108,109] and Boulware et al. [27], were designed with equity as a central focus, yet subgroup analyses were uncommon. More rigorous attention to disparity reduction is needed in future multilevel trials, including both study design and analytic strategies that examine differential effects across social groups.

While multilevel interventions are promising avenues for health promotion, it is possible benefits are not similar across all participants. Few moderators of intervention effectiveness were examined among the reviewed studies, where we found promising evidence that multilevel interventions may be more effective for those with higher baseline BP. This partially replicates previous findings that lifestyle-based interventions may have differential treatment effects depending on one’s baseline health [20]. Compared to our findings, however, previous work suggests that pharmacologic interventions have similar CVD risk-reducing effects regardless of one’s baseline risk [21,22]. This discrepancy may be driven by some multilevel interventions addressing both upstream contributors to CVH and pharmacotherapy in tandem rather than pharmacotherapy alone. For example, healthcare workers may have improved patients’ self-empowerment around disease management and/or physician-patient communication, in turn improving patients’ treatment adherence and driving improved BP. As most interventions involved individual-level activities, it is possible those with poorer baseline BP had more treatment options and thus greater engagement with the intervention.

Although none of the interventions were explicitly designed for those with multiple chronic conditions, several interventions were conducted among those with type 2 diabetes, a condition with significantly overlapping risk factors with hypertension [23]. Compared to other disease management guidelines (e.g., Joint National Committee-8 for hypertension [24]; Kidney Disease-Improving Global Outcomes for chronic kidney disease [25]), the American Diabetes’ Association provides standards of care for those with comorbid hypertension [26]. Given the large proportion of Americans with multiple chronic health conditions [27], treating common underlying risk factors together rather than the separate health conditions may improve health outcomes and healthcare [28], especially for those with complex medical needs. Future research should develop, implement, and evaluate innovative models of care, such as multilevel interventions, to prevent and manage multiple chronic conditions [119].

Despite the promise of multilevel interventions, few studies reported formal economic evaluations, limiting our understanding of their scalability and sustainability. A small number of trials assessed cost-effectiveness or cost-related outcomes. For example, Wang et al. evaluated the economic impact of telephone-based self-management interventions for blood pressure control and found them to be cost-effective relative to usual care [81]. Similarly, the PEP4PA trial assessed both health outcomes and cost-effectiveness of a multilevel physical activity intervention in low-income older adults [32]. Additionally, Katon et al. conducted a cost-effectiveness analysis of a collaborative care intervention for patients with depression and poorly controlled diabetes and/or coronary heart disease, including hypertension, and found it to be cost-effective over a 24-month period [52]. However, the vast majority of trials did not report resource use or economic implications. This gap in the literature poses challenges for broader implementation, particularly in under-resourced settings. Future studies should integrate economic evaluations alongside clinical outcomes to better assess the value and feasibility of these interventions in real-world contexts.

Finally, attention should be drawn to the continued lack of rural community interventions for hypertension management. It is known that hypertension rates in rural areas are roughly 10% higher than urban areas [120]. Studies with community interventions included in this review did address the highest at-risk population for hypertension (non-Hispanic Black adults) but were generally located in urban community settings (e.g., churches and barbershops). While interventions were generally successful, future work should focus on implementation of these interventions in the rural setting. Hypertension intervention work has begun emphasizing underserved or resource-limited populations, but many studies are still situated in urban settings [121,122]. Recently, the AHA has identified the rural population as one that needs researchers’ attention [123]. Incorporating the findings of this review and the intervention strategies highlighted is imperative to improve hypertension management in this vulnerable population.

This review has several limitations. As a rapid review, we used streamlined methods to balance rigor and efficiency. While individual study risk of bias was assessed using the Cochrane RoB 2.0 tool, we did not conduct a formal certainty of evidence appraisal (e.g., GRADE), which is consistent with the methodological scope of rapid reviews. A formal GRADE approach would have allowed for a more structured appraisal of overall confidence in the body of evidence and strength of recommendations. Additionally, the search was limited to peer-reviewed, English-language publications and U.S.-based samples, which may have excluded relevant studies. Future systematic reviews could expand on this work by incorporating such tools and broader inclusion criteria to evaluate the strength and certainty of evidence more comprehensively.

## 11. Conclusions

Multilevel interventions to improve BP among adults in the U.S. are markedly variable but predominantly involve the participant/patient and healthcare team. While these interventions show potential for improving health, it is not consistent across trials and remains unclear which combination(s) produce the most health benefits. Given the saltatory effects were not maintained, it is also important for researchers to consider the long-term sustainability of any multilevel intervention they develop. Additionally, most of the studies included in this review were conducted before the COVID-19 pandemic. This presents an important opportunity for future research to explore how multilevel interventions can be augmented to address challenges posed by the pandemic. For instance, technology-delivered components, such as telehealth or app-based interventions, may enhance accessibility and scalability while addressing barriers to in-person care. Understanding the effectiveness of these adaptations in diverse populations, particularly minoritized groups, could provide critical insights for improving BP control in the post-pandemic era. Lastly, future research should continue developing and evaluating interventions that improve both the individual and the social, physical, and structural environments in which they work and play.

## Figures and Tables

**Table 1:** Major inclusion criteria.

	Inclusion Criteria
Population	-Community-dwelling adults 18+ (e.g., not in long-term nursing) -Study conducted in the United States -Participants can come from cohort studies or among community-dwelling patient populations (e.g., adults with hypertension)
Interventions	-Multilevel interventions (any that target at least 2 levels, such as individuals and their healthcare providers) with reported results -Individual or cluster-randomized trial Multilevel: Policies, social interventions, or care teams that involve multiple people or systems. Most unilevel interventions tend to target the patient, e.g., disease self-management or psychoeducation alone or are cultural adaptations of patient-only interventions (e.g., physical activity intervention)
Comparison	Active control; no-contact control; wait-list control; treatment as usual
Outcomes	-Blood pressure (primary) -Hypertension (prevalence; risk; secondary outcomes to be reported if enough studies meet criteria) -Note: Results could include raw scores in mmHg, classification into hypertension categories, or as part of risk calculators like Life’s Essential 8 or its predecessor, Life’s Simple 7
Article-Specific Criteria	-Any publication date -Articles published in English -Published in peer-reviewed journals (i.e., not grey literature)

**Table 2:** Study-level characteristics (N = 95 manuscripts reflecting 89 unique trials) and Total Risk of Bias Score.

First Author (Year)	Sample Size and Major Demographic Characteristics	Major Inclusion Criteria	Risk of Bias Score
Individual and Healthcare Team			
Adair (2013) [19]	2135 participants across 6 clinics -9.5% non-Caucasian -48.5% men -Baseline systolic BP: 128.8 mmHg -Baseline diastolic BP: 74.4 mmHg	Adults aged 18–79 years with hypertension, diabetes, or congestive heart failure	Some Concerns
Allen (2011) [20]	525 participants -79.4% Black/African American -28.8% men Baseline systolic BP: 139.2 mmHg Baseline diastolic BP: 82.7 mmHg	Black/African American or White adults aged 21+ Diagnosed cardiovascular disease	Low
Anderegg (2018) [21]	335 participants across 32 medical offices in 15 states -62% non-Caucasian -36% men -Baseline systolic BP: 147.18 mmHg -Baseline diastolic BP: 81.26 mmHg	English- or Spanish-speaking adults 18+ with diagnosed hypertension Uncontrolled hypertension (≥140/90 or ≥130/80 with type 2 diabetes or chronic kidney disease)	Low
Bogden (1998) [22]	95 participants -25% mixed Hawaiian -42% men -Baseline systolic BP: 155.5 mmHg -Baseline diastolic BP: 95.5 mmHg	Uncontrolled hypertension (≥140/90 with target organ damage and CVD risk factors OR ≥ 150/95 without target organ damage/CVD risk factors) on 3 consecutive clinic visits in the previous 6 months	Low
Bogner (2008) [23]	64 participants -82.8% Black/African American -23% men -Baseline systolic BP: 144.9 mmHg -Baseline diastolic BP: 82.2 mmHg	Aged 50+ Presence of elevated BP and a diagnosis of depression or antidepressant prescription within past year	Some Concerns
Bogner (2013) [24]	60 participants -38.3% Black/African American -35% men -Baseline systolic BP: 136.5 mmHg -Baseline diastolic BP: 76.4 mmHg	Aged 40+ Presence of elevated BP and a diagnosis of depression or antidepressant prescription within past year	Low
Bosworth (2009) [25]	32 primary care providers and 588 patients -40% Black/African American -98% men -Baseline systolic BP: 140 mmHg -Baseline diastolic BP: 76 mmHg	Patients enrolled at Veterans Affairs Medical Center Diagnosis of hypertension by outpatient diagnostic code and had filled prescription for hypertensive medication in past year	Low
Bosworth (2018) [26]	428 participants -50% Black/African American -84.8% men -Baseline systolic BP: 130.1 mmHg -Baseline diastolic BP: 75.8 mmHg	Aged 40+ Had diagnosis of hypertension or hypercholesterolemia Poorly controlled hypertension in past year	Low
Boulware (2020) [27]	-159 participants -100% Black/African American -26.4% men -Baseline (median) systolic BP: 137 mmHg -Baseline (median) diastolic BP: 80 mmHg	English-speaking adults aged 18+ Self-reported Black/African American race Uncontrolled hypertension as evidenced by clinic records	Low
Carrasquillo (2017) [28]	300 participants -100% Hispanic/Latinx -45% men -Baseline systolic BP: 133 mmHg -Diastolic BP not reported	Hispanic/Latinx adults aged 18–65 HbA_1c_ levels of 8.0 or greater	Some Concerns
Cheng (2018) [29]	404 participants -60% men -14.6% non-Hispanic/Latinx Black/African American -68% Hispanic/Latinx -Baseline systolic BP: 149.9 Diastolic BP not reported	Participant had TIA or ischemic stroke within the past 90 days and systolic BP > 120 mm/Hg	Low
Chwastiak (2018) [30]	35 adults -66% men -60% non-White -Baseline systolic BP: 134.9 mmHg -Diastolic BP not reported	Participants with psychosis HbA_1c_ levels of 8.0 or greater Presence of diabetes	Low
Cooper (2011) [31]	41 primary care physicians and 279 patients Physician Demographics -46% men -29% Black/African American Patient Demographics -34% men -62% Black/African American -Baseline systolic BP: 135.2 mmHg -Baseline diastolic BP: 75.6 mmHg	General internists and family physicians who saw patients at least 20 h/week Patients were aged 18+ and had a diagnosis of hypertension	Some Concerns
Crist (2022) [32]	476 participants across 12 senior or community centers -24.3% men -62.4% Black/African American -Baseline systolic BP: 129.7 mmHg -Baseline diastolic BP: 71.9 mmHg	Senior/community centers that primarily served low-income populations and offer at least 1 physical activity class Participants had to be at least 50 years old and able to walk without human assistance	Low
Crowley (2013) [33]	359 adults across 2 clinics -28% men -100% Black/African American -Baseline systolic BP: 136.8 mmHg -Diastolic BP not reported	Black/African American adults aged 18+ with type 2 diabetes	Low
Crowley (2016) [34]	50 participants -96% men -54% Black/African American -Baseline systolic BP: 127 mmHg -Baseline diastolic BP: 80 mmHg	Veteran with type 2 diabetes and HbA_1c_ levels of 9.0 or greater for >year	Some Concerns
Daumit (2020) [35]	269 participants -47.6% men -46% Black/African American -Baseline systolic BP: 119.5 mmHg -Baseline diastolic BP: 75.1 mmHg	Adults aged 18+ with hypertension, diabetes, dyslipidemia, current tobacco smoking, and/or overweight/obese Participants received care from outpatient psychiatric rehab program	Low
De La Rosa (2020) [36]	6 nursing student case managers and 58 participants -Gender and race not reported -Baseline systolic BP: 135 mmHg -Baseline diastolic BP: 82 mmHg	Patients with diabetes	Some Concerns
Dennison (2007) [37] Hill (2003) [38]: 36-month outcomes	309 adults -100% men -100% Black/African American -Baseline systolic BP: 147.5 mmHg -Baseline diastolic BP: 99 mmHg	Black/African American men between 21 and 54 years old and had hypertension	Some Concerns
Emery-Tiburcio (2019) [39]	250 participants -50% Black/African American -50% Hispanic/Latinx -19.6% men -Baseline systolic BP: 140.6 mmHg -Baseline diastolic BP: 77.2 mmHg	Self-identified Black/African American adults aged 60+ BMI > 25.0 and symptoms of depression as evinced by Patient Health Questionnaire-9 scores ≥ 8	Low
Feldman (2016) [40]	845 participants and 312 nurses -100% Black/African American -34% men -Baseline systolic BP: 155.5 mmHg -Baseline diastolic BP: 87.3 mmHg	Black/African American adults with uncontrolled hypertension	Some Concerns
Gabbay (2006) [41]	332 participants -No race reported -55% men -Baseline systolic BP: 136.5 mmHg -Baseline diastolic BP: 77 mmHg	Participants with diabetes and aged 18+	Low
Gabbay (2013) [42]	545 participants -46.5% non-White -38.7% Hispanic/Latinx -41.8% men -Baseline systolic BP: 143.5 mmHg -Baseline diastolic BP: 79 mmHg	Participants aged between 18 and 75 with type 2 diabetes and had one of the following: (1) HbA_1c_ levels ≥ 8.5%; (2) BP > 140/90 mmHg; or (3) LDL > 130	Some Concerns
Gary (2003) [43]	186 participants -100% Black/African American -24% men -Baseline systolic BP: 127 mmHg -Baseline diastolic BP: 76 mmHg	Black/African American adults aged between 35–75 Presence of type 2 diabetes	Low
Green (2014) [44]	101 participants -5% Black/African American -58% men -Baseline systolic BP: 150.4 mmHg -Baseline diastolic BP: 91.6 mmHg	Adults aged 30–69 years old with BMI > 26, elevated BP, and 10–25% 10-year Framingham CVD risk	Some Concerns
Heisler (2012) [45]	4100 participants across 16 primary care teams at 5 medical centers -70% men -15% Black/African American -10% Hispanic/Latinx -Systolic BP from last 3 months: 156.5 mmHg -Diastolic BP from last 3 months: 79 mmHg	Patients with diabetes Evidence of persistent poor BP control, poor medication refill adherence, or insufficient medication intensification	Low
Hennessy (2006) [46]	10,698 participants across 93 providers -43.1% men -41% Black/African American -53% with baseline BP < 140/90 mmHg	Physicians and nurse practitioners in family medicine, internal medicine, and obstetrics-gynecology who cared for at least 10 eligible participants Participants had a diagnosis of hypertension (ICD-9 codes of 401.x or 402.x)	Some Concerns
Hiss (2007) [47]	197 participants -33% men -27% Black/African American -Baseline systolic BP: 131.2 mmHg -Baseline diastolic BP: 73.9 mmHg	Adult participants with type 2 diabetes	Some Concerns
Hunt (2008) [48]	463 participants -35% men -Race not reported -Baseline systolic BP: 173.5 mmHg -Baseline diastolic BP: 91 mmHg	Participants with known hypertension and uncontrolled BP Participants who have received care at study practice	Low
Islam (2023) [49]	303 participants -49.5% men -100% South Asian -Baseline systolic BP: 138.2 mmHg -Baseline diastolic BP: 86.4 mmHg	Self-reported South Asian ancestry from India, Bangladesh, Pakistan, Nepal, Sri Lanka, or the Indo-Caribbean Aged between 18 and 85 Diagnosis of hypertension and/or uncontrolled BP within the past 6 months	Low
Kangovi (2017) [50]	302 participants -24.5% men -95% Black/African American -Baseline systolic BP: 143.8 mmHg -No diastolic BP reported	Participants living in a high-poverty region of Philadelphia Presence of 2 or more following chronic diseases: hypertension, diabetes, obesity, tobacco dependence	Low
Katon (2010) [51] Katon (2012) [52]	214 participants -48% men -47% non-White or Hispanic/Latinx -Baseline systolic BP: 134 -No diastolic BP reported	Participants with diagnosis of diabetes, coronary heart disease, or both Patents had one or more measure of poor disease control within past 112 months	Low Low
Kilbourne (2013) [53]	116 participants -83% men -5% non-White -Baseline systolic BP: 132.8 mmHg -Baseline diastolic BP: 82.2 mmHg	IDC-9 diagnosis of bipolar disorder ≥1 cardiovascular disease risk factor	Some Concerns
Krein (2004) [54]	246 participants -97% men -41.7% non-White -Baseline systolic BP: 145 mmHg -Baseline diastolic BP: 86 mmHg	Adults aged 18+ who were diagnosed with type 2 diabetes and had HbA_1c_ levels ≥ 7.5%	Low
Lin (2014) [55]	214 adults -52% men -No race reported -Baseline systolic BP range: 93.5–195.5 mmHg -No diastolic BP reported	Participants with depression and uncontrolled diabetes and/or coronary heart disease	Low
Ma (2009) [56]	419 adults -34.4% men -9.6% Black/African American -63% Hispanic/Latinx -Baseline systolic BP: 133.9 mmHg -Baseline diastolic BP: 79.6 mmHg	Adults between 35 and 85 years old who had moderately to-severely elevated levels of major modifiable cardiovascular disease risk factors	Low
Margolis (2018) [57]	450 participants across 16 clinics -55% men -12% Black/African American -Baseline systolic BP: 148 mmHg -Baseline diastolic BP: 84.7 mmHg	Individuals with a BP of 140/90 mmHg or high at 2 most recent primary care encounters in the last year Uncontrolled BP obtained from 3 measures in a research clinic	Low
Margolis (2022) [58]	3071 participants across 21 primary care clinics -47% men -19% Black/African American -Baseline systolic BP: 158.0 mmHg -Baseline diastolic BP: 91.7 mmHg	Participants aged between 18 and 85 years who had an encounter with one of the study clinic sites during the enrollment period and had a hypertension diagnosis code within the past 24 months	Some Concerns
McClintock (2017) [59]	54 participants from 3 primary care clinics -26% men -59% Black/African American -Baseline systolic BP: 134.3 mmHg -Baseline diastolic BP: 75.25 mmHg	Participants aged 18+ with a diagnosis of hypertension and a current prescription for an antihypertensive	Some Concerns
Nguyen-Huynh (2022) [60]	1761 participants -31% men -100% Black/African American -Baseline systolic BP: 150.5 mmHg -Baseline diastolic BP: 84.5 mmHg	Self-reported Black/African American adults aged 18+ who were listed in a hypertension registry	Some Concerns
Planas (2012) [61]	65 participants -41% men -15% Black/African American -Baseline systolic BP: 140.1 mmHg -Baseline diastolic BP: 76.7 mmHg	Adults aged 18+ and had a most recent recording of HbA_1c_ levels of 7.0% or greater	Some Concerns
Prezio (2013) [62]	180 participants -39% men -81.7% Hispanic/Latinx -11.7% Black/African American -Baseline systolic BP: 126 mmHg -Baseline diastolic BP: 79 mmHg	Active clinic patients between the ages of 18–85 years old and diagnosed with type 2 diabetes	Low
Rosas (2015) [63]	207 participants -76.8% men -100% Hispanic/Latinx -Baseline systolic BP: 115.2 mmHg -Baseline diastolic BP: 73.6 mmHg	Spanish-speaking Hispanic/Latinx adults BMI between 30 and 60 and one or coronary heart disease risk factors	Low
Scott (2006) [64]	149 participants -38.9% men -3.4% Black/African American -32.2% Hispanic/Latinx -Baseline systolic BP: 130.5 mmHg -Baseline diastolic BP: 79.4 mmHg	Adults aged 18+ with a diagnosis of type 2 diabetes	High
Vaughan (2017) [65]	50 participants Gender not reported -100% Hispanic/Latinx -Baseline systolic BP: 133.5 mmHg -Baseline diastolic BP: 81.6 mmHg	Hispanic/Latinx adults aged 18+ with a documented diagnosis of type 2 diabetes or prediabetes	Some Concerns
Von Korff (2011) [66]	214 participants across 14 care clinics -48% men -23% non-White -Baseline systolic BP: 134 mmHg -Diastolic BP not reported	Participants with diabetes and/or coronary heart disease; evidence of poor BP, cholesterol, or glycemic control	Low
Zillich (2005) [67]	125 participants across 12 community pharmacies -2% non-White -39.2% men -Baseline systolic BP: 151.5 mmHg -Baseline diastolic BP: 85.3 mmHg	Participants were aged 21+ with a diagnosis of hypertension, took 1–3 BP medications with no recent changes (past 4 weeks), and had uncontrolled BP	Low
Individual and Healthcare Team and Health Policies			
Holtrop (2017) [68]	1392 participants across 10 practices -53.8% men -Race not reported -Baseline systolic BP: 127.3 mmHg -No diastolic BP reported	Adults aged 18+ receiving care at one of the study practices A diagnosis of type 2 diabetes or obesity (BMI ≥ 30)	Some Concerns
Huebschmann (2012) [69]	591 participants -45.4% men -23.2% Black/African American -Baseline systolic BP: 149 mmHg -Baseline diastolic BP: 90.5 mmHg	Participants aged 18–79 years with elevated BP based on 2 or 3 most recent clinic visits	Some Concerns
Murray (2004) [70]	712 participants -56% men -33% Black/African American -Baseline systolic BP: 143 mmHg -Baseline diastolic BP: 76.75 mmHg	Participants had an active outpatient diagnosis of hypertension or (1) at least 2 systolic BP measurements of 140 mmHg or greater, (2) 2 diastolic BP measurements of 90 mmHg or greater, and (3) a prescription for at least one antihypertensive medication	Some Concerns
Piatt (2010) [71]	119 participants across 11 primary care practices -50% men -8% non-White -Baseline systolic BP: 141.1 mmHg -Baseline diastolic BP: 74.6 mmHg	Participants with diagnosis of type 2 diabetes receiving care from one of the study sites	Some Concerns
Ralston (2009) [72]	83 participants -51.6% men -18.7% non-White -Baseline systolic BP: 133 mmHg -Baseline diastolic BP: 76 mmHg	Adults aged between 18 and 75 and had a most recent recording of HbA_1c_ levels of 7.0% or greater in the past 12 months	Low
Rothman (2005) [73]	217 participants -64% Black/African American -44% men -Baseline systolic BP: 138.5 mmHg -Baseline diastolic BP: 81 mmHg	Adults aged 18+ with a clinical diagnosis of type 2 diabetes Those with poor glycemic control (HbA_1c_ levels of 8.0% or more)	Low
Roumie (2006) [74]	1341 participants across 182 providers -Race and gender not reported -Baseline systolic BP: 157.2 mmHg -Baseline diastolic BP: 82.7 mmHg	Adults aged between 21 and 90 years, filled their medications at Veterans Administration pharmacies, were only taking 1 antihypertensive medication, and had at least 2 uncontrolled BP measures in 6-month baseline period.	Some Concerns
Schoenthaler (2020) [75]	119 participants -50.4% men -100% Hispanic/Latinx -Baseline systolic BP: 141.7 mmHg -Baseline diastolic BP:81.2 mmHg	Self-identified Hispanic/Latinx adults aged 18+ with uncontrolled hypertension, taking at least 1 antihypertensive medication and were nonadherent, and had at least 1 comorbid diagnosis	Low
Svarstad (2013) [76]	576 participants across 28 chain pharmacies -33.9% men -100% Black/African American -Baseline systolic BP: 152.2 mmHg -Baseline diastolic BP: 92.5 mmHg	Patients were eligible if they were Black/African American adults aged 18+, had an active antihypertensive medication filled at the study pharmacy, and presence of elevated BP	Low
Svetkey (2009) [77]	574 participants and 32 physicians -39% men -37% Black/African American -Baseline systolic BP: 133.1 mmHg -Baseline diastolic BP: 74.1 mmHg	Patients were eligible if they were 25+ and had hypertension based on billing codes	Low
Talavera (2021) [78]	456 participants -36.3% men -100% Hispanic/Latinx -Baseline systolic BP: 128 mmHg -Baseline diastolic BP: 74.6 mmHg	Self-identified Hispanic/Latinx adults aged 18+ with physician diagnosis of type 2 diabetes and no current insulin use	Low
Towfighi (2021) [79]	487 participants -65.1% men -18.3% Black/African American -71.3% Hispanic/Latinx -Baseline systolic BP: 144.4 mmHg -No diastolic BP reported	Adults aged 40+ who experienced ischemic, hemorrhagic stroke, or transient ischemic attack within the past 90 days; elevated systolic BP	Low
Vinicor (1987) [80]	509 participants -22% men -71.3% Black/African American -Baseline systolic BP: 140 mmHg -Baseline diastolic BP: 82.7 mmHg	Participants with type 2 diabetes	Some Concerns
Wang (2012) [81]	591 participants -92% men -48% Black/African American -Baseline systolic BP: 129 mmHg -Baseline diastolic BP: 77 mmHg	Inadequate BP control in year before enrolling in study trial	Low
Welch (2011) [82]	46 participants -35% men -100% Hispanic/Latinx Race not reported -Baseline systolic BP: 137.5 mmHg -Baseline diastolic BP: 80.5 mmHg	Adults aged between 30 and 85 years old Presence of type 2 diabetes for at least 1 year based on medical review and treatment history Hispanic/Latinx ethnicity HbA_1c_ levels between 7.5–14%	Low
Individual and Community Environment			
Ard (2017) [83]	409 participants across 8 counties -100% Black/African American -0% men -Baseline systolic BP: 125.1 mmHg -Baseline diastolic BP: 79.5 mmHg	Self-identified Black/African American women aged between 30 and 70 years Overweight or obese (BMI ≥ 25 kg/m^2^)	Low
Brewer (2022) [84]	136 participants across 16 churches -100% Black/African American -29% men -Baseline systolic BP: 134.31 mmHg -Baseline diastolic BP: 81.38 mmHg	Churches with predominantly Black/African American parishioners	Low
Brown (2015) [85]	760 participants across 10 churches -83.7% Hispanic/Latinx -36.2% men -Baseline systolic BP: 125.5 mmHg -Baseline diastolic BP: 79.2 mmHg	Hispanic/Latinx or non-Hispanic/Latinx White adults aged 18+ Parishioner of participating parish	Some Concerns
Derose (2019) [86]	268 adults across 4 churches (Baptist and Catholic) -45.3% Black/African American -51.1% Hispanic/Latinx -25.4% men -Baseline systolic BP: 131.9 mmHg -Baseline diastolic BP: 76.2 mmHg	Adults aged 18+ who attend church at least monthly	Some Concerns
Kerr (2018) [87]	307 participants across 11 San Diego retirement communities -27% men -No race reported -Baseline systolic BP: 131.4 mmHg -Baseline diastolic BP: 68.1 mmHg	Adults aged 65+ Retirement communities with at least 100 residents, had independent living options, and had stores or parks within 1 mile	Low
Nafziger (2001) [88]	626 participants -44.6% men -1% non-White -Baseline systolic BP: 127.2 mmHg -Baseline diastolic BP: 78.0 mmHg	Adults aged between 20 and 69 years and lived in the study counties (rural New York) for at least 6 months of the year	Some Concerns
Paskett (2018) [89]	663 participants across 60 churches in 5 states -29.3% men -1.8% Black/African American -Baseline systolic BP: 135.9 mmHg -Baseline diastolic BP: 82.4 mmHg	Churches with >250 members in Appalachian counties interested in participating in the study Participants at least 18 years old and attended church at least 4 times in the past 2 months BMI of at least 25	Low
Seguin (2018) [90]	194 participants -0% men -5% non-White -Baseline systolic BP: 134.4 mmHg -Baseline diastolic BP: 87.5 mmHg	Women aged 40+ with BMI ≥ 25 and sedentary or not meeting physical activity guidelines	Low
Seguin-Fowler (2020) [91]	182 participants -0% men -2.4% non-White -Baseline systolic BP: 132.6 mmHg -Baseline diastolic BP: 85.4 mmHg	Women aged 40+ with BMI between 25 and 30 and sedentary or not meeting physical activity guidelines	Low
Individual and Family/Social Support			
Baranowski (1990) [92]	94 Black/African American families -100% Black/African American -No baseline BP reported	Families with at least one self-reported Black/African American child between the 5th and 7th grade No diagnosed cardiovascular disease	Low
Matthan (2022) [93]	205 participants -6% men -17.4% Black/African American -74.8% Hispanic/Latinx -67% with normal BP (<120/80 mmHg)	Children between the ages of 7–12 years and BMI ≥ 85th percentile for age and sex	Low
Rosland (2022) [94]	239 participants and care partner dyads -96.7% men -12.6% Black/African American -Baseline systolic BP: 140.2 mmHg -Diastolic BP not reported	Adults aged between 30 and 70 years with type 2 diabetes Those with poor glycemic or BP control during past 9 months	Low
Trief (2016) [95] Trief (2019) [96]	268 participants -61.6% men -17.3% Black/African American -7.1% Hispanic/Latinx -Baseline systolic BP: 126.7 mmHg -Baseline diastolic BP: 74.4 mmHg 268 care partners -35.4% men -16.1% Black/African American -6.1% Hispanic/Latinx -Baseline systolic BP: 122.8 mmHg -Baseline diastolic BP: 73.8 mmHg	Patients were adults aged 21+ who had diagnosis of type 2 diabetes for >1 year and baseline HbA_1c_ levels ≥ 7.5%; and in a self-defined committed relationship for ≥1 year	Low Low
Wieland (2018) [97]	151 participants (81 adolescents and 70 adults—adult values presented here) -28.6% men -39% Somali or Sudanese -61% Hispanic/Latinx -Baseline systolic BP: 122.1 mmHg -Baseline diastolic BP: 76.3 mmHg	Households with at least 1 adult and 1 adolescent aged 10–18 years	Low
Yates (2015) [98]	34 participants and their spouses/partners -14.8% men -6% Hispanic/Latinx -6% non-White -Baseline systolic BP: 126 mmHg -No diastolic BP reported	Participants aged 19+ who are the spouse/partner (>1 year) of someone who received coronary artery bypass surgery For these analyses, only the spouses/partners were included.	Low
Individual and Family/Social Support and Healthcare Team			
Haskell (2006) [99]	148 participants -7% Black/African American -57% Hispanic/Latinx -43.3% men -Baseline systolic BP: 141 mmHg -Baseline diastolic BP: 82 mmHg	Adults aged between 35 and 80 years old who had (1) limited or no health insurance and low family income, (2) increased risk of having a cardiac event, and (3) currently received medical care at nonprofit or free clinic/hospital	Low
Levine (2003) [100]	789 participants -38.15% men -100% Black/African American -Baseline systolic BP: 148.15 mmHg -Baseline diastolic BP: 89.2 mmHg	Black/African American adults aged 18+ with hypertension	Low
Pearce (2008) [101]	199 participants and 108 support persons across 18 practices -44.7% men -13.1% Black/African American -Baseline systolic BP: 140.3 mmHg -Baseline diastolic BP: 76.8 mmHg	Adults aged 21+ Participant had type 2 diabetes based on chart review, diagnosis, or a current prescription for antidiabetic drugs Hypertension with suboptimal control	Low
Pérez-Escamilla (2015) [102]	211 participants -26.5% men 100% Hispanic/Latinx -Baseline systolic BP: 119.5 mmHg -No diastolic BP reported	Self-identified Hispanic/Latinx adults aged 21+ with a documented diagnosis of type 2 diabetes for >12 months, had HbA_1c_ levels of 7.0% or greater	Low
Healthcare Team and Health Policies			
Petersen (2013) [103]	77 physicians across 12 hospitals 56% men -7.7% Black/African American -80% of patients with controlled hypertension at baseline	Inclusion criteria not specified	Low
Peterson (2008) [104]	7101 participants across 24 practices -50% men -Race not reported -Baseline systolic BP: 132.75 mmHg -No diastolic BP reported	Major practice-level eligibility: (1) single-specialty community primary care, (2) availability of 24 months of billing data, (3) 3–22 full-time equivalent providers Major participant eligibility: Adults aged between 18 and 89 years old with type 2 diabetes	Low
Weber (2010) [105]	179 participants -43% men -9% non-White -Baseline systolic BP: 151.7 mmHg -Baseline diastolic BP: 85.1 mmHg	Adults aged between 21 and 85 years receive between 0 and 3 antihypertensive agents with no changes to their regimen within the past 4 weeks Clinic systolic BP between 145 and 179 mmHg or diastolic BP of 95–109 mmHg (without diabetes) Clinic systolic BP between 135 and 179 mmHg or diastolic BP of 85–109 mmHg (with diabetes)	Low
Individual and Healthcare Team and Community Environment			
Victor (2011) [106] Rader (2013) [107] subgroup analysis	1297 participants across 17 barbershops in Dallas County -100% men -100% Black/African American -Baseline systolic BP: 137 mmHg -Baseline diastolic BP: 80 mmHg 138 participants -100% men -100% Black/African American -Baseline systolic BP: 157 mmHg -Baseline diastolic BP: 90 mmHg	Barbershops had non-Hispanic/Latinx Black/African American owners and barbers, had been in business for 10+ years, had 3 or more barbers, >90% Black/African American men clientele Customers eligible for the intervention were self-reported non-Hispanic/Latinx Black/African American men aged 18+. (Rader) This was a subgroup analysis limited to those with baseline systolic BP ≥ 140 mmHg and 10-month follow-up data	Low Low
Victor (2018) [108] Victor (2019) [109]	319 participants across 52 barbershops in Los Angeles County -100% men -100% Black/African American -Baseline systolic BP: 153.7 mmHg -Baseline diastolic BP: 91 mmHg	Non-Hispanic/Latinx men aged between 35 and 79 years old with systolic BP of 140 mmHg	Low Low
Individual and Community Environment and Health Policies			
Pereira (2020) [110]	630 participants across 24 work sites -4.1% Black/African American -13.8% Hispanic/Latinx -Baseline systolic BP: 124.3 mmHg -Baseline diastolic BP: 77.5 mmHg	Full-time employees of study work site aged 18+, have generally good health, and willing to have a sit–stand workstation installed Worksites had 20–60 employees with predominantly seated desk-based office work who did not have a current worksite wellness program	Low
Wilcox (2013) [111]	1257 participants across 128 churches -24.3% men -99.4% Black/African American -Baseline systolic BP: 128.5 mmHg -Baseline diastolic BP: 70.6 mmHg	Participants aged 18+ who were free of serious medical conditions that would make physical activity or diet changes difficult Participants needed to attend church services at least 1 time/month	Low
Individual and Health Policies			
Skolarus (2023) [112]	488 participants -22.8% men -54% Black/African American -Baseline systolic BP: 146 mmHg -No diastolic BP reported	Participants with at least 1 documented systolic BP ≥ 160 mmHg or a diastolic BP ≥ 90 mmHg, likely to be discharged from the emergency department, and had text messaging capability	Some Concerns
Individual and Family/Social Support and Healthcare Team and Community Environment			
Campbell (2014) [113]	111 children from 109 families -47% men -96% Black/African American -No baseline BP reported	-Children born between 1972–1977 -High-Risk Index > 11 to indicate disadvantaged background	Some Concerns

Note: BP = Blood pressure.

**Table 3:** Intervention activities and summary of results.

First Author (Year) Intervention Duration	Intervention Activities	Control Group	Summary of Results
Individual and Healthcare Team			
Adair (2013) [19] 12 months	Individual: Participate in individualized care plan activities Healthcare Team: Nonclinical care guide works with participant, physician to address care goals; submit quarterly reports to physician on goal progress and participant concerns	Usual care	Significantly more treatment goals were met in the intervention compared to the usual care group
Allen (2011) [20] 12 months	Individual: Therapeutic lifestyle changes, including diet, exercise, and smoking cessation Healthcare Team: Nurse practitioner and community health worker teams for lifestyle counseling, medication titration/prescription, and communicate goals with physician	Enhanced usual care	Significant reductions compared to control group in systolic BP (6.2 mmHg) and diastolic BP (3.1 mmHg); Systolic BP reductions were clinically significant
Anderegg (2018) [21] 9 months	Individual: Participate in individualized care plan activities Healthcare Team: Pharmacists co-developed individualized care plan for BP goal with participant; care plan was discussed and adjusted with physician input	Usual care	Significant reductions compared to control group in systolic BP (8.64 mmHg) and diastolic BP (2.90 mmHg)
Bogden (1998) [22] 6 months	Individual: Participate in individualized care plan activities Healthcare Team: Pharmacists, physicians, and patients would coordinate a treatment regimen in shared medical appointments	Usual care	Significant decrease in systolic BP (14 mmHg) and diastolic BP (23 mmHg) in the intervention; BP changes were greater in intervention group Intervention effects unrelated to age, gender; effective for mixed-ancestry Hawaiians.
Bogner (2008) [23] 4 weeks	Individual: Participate in individualized care plan activities Healthcare Team: Integrated care manager who provided patient with individualized hypertension and depression program; collaborated with physician to deliver care and monitor adherence	Usual care	Significantly greater decreases in posttest systolic BP and diastolic BP among intervention group (127.3 mmHg and 75.8 mmHg, respectively).
Bogner (2013) [24] 12 weeks	Individual: Participate in individualized care plan activities Healthcare Team: Integrated Licensed Practical Nurses who provided patient with individualized hypertension and depression program; collaborated with physician to deliver care and monitor adherence	Usual care	Significantly greater decreases in posttest diastolic BP (74.2 mmHg) but not systolic BP (130 mmHg) among intervention relative to control group
Bosworth (2009) [25] 24 months	Individual: Participate in individualized educational and behavioral intervention with nurse Healthcare Team: Computer-assisted medication decision support system for physicians; quarterly audit and feedback of entire patient panel regarding BP targets, medication choices	Usual care (clinical reminders); intervention was factorial design	No significant posttest differences in participants’ systolic BP by intervention group.
Bosworth (2018) [26] 12 months	Individual: Participate in disease self-management activities; individualized educational and behavioral intervention Healthcare Team: Clinical pharmacist-led behavioral intervention for patients; coordinate with physician to adjust medication as needed	Education control group	Both groups had significantly decreased systolic BP over time; No significant posttest differences in participants’ systolic BP or diastolic BP by intervention group
Boulware (2020) [27] 12 months	Individual: Participate in hypertension self-management activities; 9 weeks of problem-solving training Healthcare Team: Community health workers provided training to participants; address barriers to hypertension care (including resource linking); communicate with clinic nurses if BP was not under control	Usual care (single contact with community health workers to receive BP monitor)	Systolic and diastolic BP improved across time in all groups; there were no significant intervention effects
Carrasquillo (2017) [28] 12 months	Individual: Participate in group psychoeducation and exercise activities Healthcare Team: Community Health Worker provided health education, patient navigation (including linking resources and serving as a liaison between patients, physicians), health coaching; assisted with nonmedical services (e.g., legal assistance, employment)	Usual care	Although there was a statistical reduction in systolic BP in multivariate models, reductions were lower than planned 8 mmHg The intervention significantly lowered systolic BP only among study completers who had a baseline systolic BP ≥ 140 mmHg
Cheng (2018) [29] 12 months	Individual: Participate in disease self-management, lifestyle modification activities Healthcare Team: Disease case management with physician assistant or nurse practitioner (with computerized decision support); delivered interventions to participants, led care coordination between appts; coordinate medication adjustment with physician	Education and usual care control	There were significant reductions in systolic BP in both groups across time, but there were no significant intervention effects (*p* = 0.55)
Chwastiak (2018) [30] 3 months	Individual: Participate in disease self-management, lifestyle modification activities Healthcare Team: Weekly systematic caseload review with collaborative care team, including nurse care managers, psychiatrists, registered nurses, and endocrinologists	Usual care	There were no significant changes in systolic BP across time, regardless of group assignment
Cooper (2011) [31] 12 months	Individual: Communication coaching delivered by community health workers Healthcare Team: Physicians communication skills program training and feedback	Usual care (factorial design)	There were no significant changes in systolic or diastolic BP by intervention group Among those with uncontrolled BP at baseline, there were larger but nonsignificant changes among all interventions compared to patient and physician minimal group
Crist (2022) [32] 24 months	Individual: Participate in physical activity goal setting, self-monitoring activities Healthcare Team: Peer health coaches led group walking; organized activities in community (e.g., “community advocacy to improve walking conditions”)	Usual care	There were significant reductions among the intervention group for systolic BP and diastolic BP at 18 months, but there were no other between-group differences. This was not sustained at 24 months.
Crowley (2013) [33] 12 months	Individual: Diabetes self-management activities Healthcare Team: Nurse-supported disease self-management support; medication management facilitation between the patient and physician	Usual care	There were no between-group differences at posttest for systolic BP (*p* = 0.11)
Crowley (2016) [34] 6 months	Individual: Diabetes self-management activities Healthcare Team: Nurse-supported disease self-management support; medication management facilitation between the patient and physician; Physician-guided depression management	Usual care	The intervention group had significantly decreased systolic BP (*p* = 0.035) and diastolic BP (*p* = 0.013) compared to control group at posttest
Daumit (2020) [35] 18 months	Individual: Participating in individualized risk reduction education and counseling activities Healthcare Team: Health coach and nurse-delivered education and counseling; collaboration with physicians, mental health staff for treatment and arranging follow-up appointments	Usual care	There were no significant effects of the intervention on systolic or diastolic BP
De La Rosa (2020) [36] 12 months	Individual: Participate in group education and physical activity Healthcare Team: Nursing student collaborates with physicians, patients to assess needs between appts, schedule specialist appts, and review charts	Usual care	Compared to the control group, a larger proportion of those in the intervention group had controlled BP (130/80 mmHg) at posttest (61.1% vs. 36.8%; *p* = 0.009) and improved BP (61.1 vs. 24.6%; *p* < 0.001) at posttest
Dennison (2007) [37] 5 years; Hill (2003) [38]: 36-month outcomes	Individual: Participate in individualized care activities (all members of family engaged by healthcare team on an individual basis) Healthcare Team: Team care including nurse practitioners, community health workers, and physicians. Included a team review of medications, connecting participant with transportation, employment support; engage/mobilize family/social support	Education control and list of community hypertension care sources	There were between-group differences in systolic BP at Years 1, 3, and 4, but these were not sustained in Year 5. There were between-group differences in diastolic BP at Years 1, 3–4, but this was not sustained in Year 5 The intervention effect was not dependent on having a physician or nurse practitioner for hypertension care
Emery-Tiburcio (2019) [39] 12 months	Individual: Complete action plan recommendations; Engage in psychotherapy (either cognitive behavioral therapy or interpersonal psychotherapy) Healthcare Team: Semi-structured assessment of mental health and cardiovascular risk followed by virtual team case review with geropsychologist, psychiatrist, social worker, chaplain, dietitian, pharmacist, occupational therapist, and primary care physician; Program coordinator would facilitate community resource engagement on behalf of participant as requested	Health education, social support, and supportive services control (“Generations” health program)	There were no significant intervention effects on systolic (*p* = 0.77) or diastolic (*p* = 0.47) BP
Feldman (2016) [40] 12 onths	Individual: “Basic intervention”: BP self-monitoring and education with JNC7 Guide; “Augmented Intervention”: Basic Intervention and enrollment in hypertension home support program Healthcare Team: Hypertension support nurse and health educator-provided medication, self-management support for patient; Coordinate care plans and augment medication with primary care physician	Usual home health services	All groups had declining systolic BP over the trial, but there were no significant intervention effects on systolic BP at the 12-month posttest
Gabbay (2006) [41] 12 months	Individual: Participate in individualized care plan activities Healthcare Team: Nurse care manager coordinated patient care with primary care physician, including referrals to other facilities, scheduling, medication, and therapeutic recommendations	Usual care	Compared to the control group, the intervention group had significantly improved systolic and diastolic BP at posttest (*p* < 0.001)
Gabbay (2013) [42] 24 months	Individual: Participate in individualized care plan activities Healthcare Team: Nurse care manager coordinated patient care with primary care physician, including referrals to other facilities, scheduling, medication, and therapeutic recommendations	Usual care	There were no significant intervention effects on systolic BP at Year 1 (*p* = 0.33) but was present in Year 2 (*p* = 0.045)
Gary (2003) [43] 24 months	Individual: Participate in individualized care plan activities Healthcare Team: Nurse care manager who coordinated care between the patient and physician (including referrals, counseling, physician feedback and prompting); Community health worker-facilitated preventive care (e.g., schedule appointments, mobilize social support, provide physician feedback); Biweekly intervention coordination meetings between nurse care manager, community health worker	Usual care (factorial design with nurse care manager, community health worker as separate components)	Compared to the control group, the nurse care manager and community health worker group had significantly improved diastolic BP at posttest (*p* < 0.042). There were no intervention effects on systolic BP
Green (2014) [44] 6 months	Individual: Participate in BP self-monitoring and management activities Healthcare Team: Dietitian-led team care, including communicating with physicians and patients about augmenting medication management	Usual care	There were no significant intervention effects on systolic BP (*p* = 0.40), diastolic BP (*p* = 0.32), CVD risk score (*p* = 0.10), or the percentage of participants with BP control (*p* = 0.16)
Heisler (2012) [45] 14 months	Individual: Participate in disease self-education and self-management activities provided by their physician Healthcare Team: Pharmacist–physician collaboration on medication treatment; pharmacist develops action plan with participant	Usual care	Both the intervention and control groups had decreasing systolic BP by about 9 mmHg 6 months after the intervention ended; there were no significant between-group differences. The systolic BP lowering occurred more rapidly among the intervention compared to control group (i.e., immediate posttest differences were greater among intervention)
Hennessy (2006) [46] 6 months	Individual: Participate in disease self-education and self-management activities provided by their physician Healthcare Team: Pharmacist-delivered education to physicians; they also received physician-specific audit reports on the proportion of those with managed hypertension and percentile rank in health system	Usual care	There were no significant intervention effects on systolic or diastolic BP
Hiss (2007) [47] 6 months	Individual: Participate in action plan for disease management Healthcare Team: Nurse care manager-led counseling for participant; Treatment plans and patient goals discussed with physician to co-develop action plan	Comprehensive diabetes evaluation and results delivered to physician	There were significant intervention effects on systolic BP (*p* = 0.0007) but not diastolic BP (*p* = 0.39) Participants with baseline systolic BP values ≥ 130 mmHg improved if they had >2 contacts with study nurse, but not if they had <2 contacts
Hunt (2008) [48] 12 months	Individual: Participate in hypertension self-management activities Healthcare Team: Pharmacist-led discussions with patient about medications, lifestyle habits, barriers to care, education, and follow-up care	Usual care	There were significant intervention effects on systolic (*p* = 0.007) and diastolic (*p* = 0.002) BP
Islam (2023) [49] 6 months	Individual: Participate in group education sessions; execute action plan as discussed with community health worker Healthcare Team: Community health workers engaged participants in participant goal-setting, medication adherence, and activity monitoring; provide resource linking (e.g., finding South Asian-serving organizations; connecting with food pantries)	One-time education lecture and usual care	Among those with posttest data, a greater proportion of those in the intervention group had controlled BP compared to the control group (68.2% vs. 41.6%, *p* < 0.001). The intervention group had 3.7 times the odds of achieving BP control at follow-up compared to the control group
Kangovi (2017) [50] 6 months	Individual: Participate in disease self-management activities to achieve individualized goals Healthcare Team: Community health workers engaged participants in participant goal-setting, medication adherence, and activity monitoring; provide resource linking (e.g., visit food pantry with participant); Community health worker-led weekly patient support group; managers provided support, training, burnout prevention training, and review patient documents with community health workers; community health workers communicated action plans + progress to physicians	Goal-setting alone and usual care	There were clinical, albeit nonsignificant posttest differences in systolic BP favoring the intervention group (11.2 mmHg vs. 1.8 mmHg decrease, *p* = 0.08)
Katon (2010) [51] 12 months; Katon (2012) [52] (18 and 24 months follow-up)	Individual: Participated in chronic disease self-management activities based on discussion with nurse–physician team Healthcare Team: Physician-nurse collaborative team to develop patient clinical and self-care goals; weekly team meetings between nurses, psychiatrists, physicians, and psychologists to review medications and clinical responses	Enhanced usual care	There were significant intervention effects on systolic BP (unadjusted between-group difference = 5.1 mmHg) at 12 months The intervention effects on systolic BP were not maintained at 18 and 24 months follow-up
Kilbourne (2013) [53] 24 months	Individual: Participate in disease self-management activities Healthcare Team: Health specialist-delivered self-management education, wellness monitoring, clinical registry tracking, and links to community resources to participant; health specialist facilitated patient-physician communication, including relaying patient health concerns; ongoing clinical management by primary care and mental health provider team; health specialist provided summary information on bipolar disorder treatment to physicians and mental health providers	Enhanced usual care (quarterly wellness newsletters and standard care)	There were significant intervention effects on systolic (*p* = 0.04) and diastolic (*p* = 0.04) BP
Krein (2004) [54] 18 months	Individual: Participate in disease self-monitoring activities based on treatment goals discussed with case manager Healthcare Team: Case manager-led training patients on disease self-management, including exercise and diet; provide reminders for recommended screening/tests; aid with appointment scheduling/referrals/and identify/initiate medication treatment/changes; physician or case manager collaborate on augmenting medication dosage	Usual care	There were no significant intervention effects on systolic (*p* = 0.53) or diastolic (*p* = 0.61) BP
Lin (2014) [55] 12 months (followed up to 24 months)	Individual: Participate in disease treatment goals developed in collaboration with nurse care managers and physicians Healthcare Team: Nurse care manager monitored clinical progress and supported patient medication adherence and lifestyle changes; weekly case reviews with nurse care manager, patients’ physicians and care team, and medical and psychiatric consultants; after achieving target levels, nurse and patient co-develop relapse prevention and maintenance plan	Usual care	Among those with poorer baseline systolic BP control (>140 mmHg), there were significant intervention effects at 6 months (*p* = 0.012), but this effect was not sustained at 12, 18, or 24 months There were no significant intervention effects for any timepoint among those with fair systolic BP control at baseline
Ma (2009) [56] 15 months	Individual: Participate in individualized treatment plan developed with case management team Healthcare Team: Nurse and dietitian-led case management integrated with patients’ primary care teams; referred participant to other resources/appointments as necessary	Usual care	There were significant intervention effects on both systolic (*p* = 0.003) and diastolic (*p* = 0.02) BP
Margolis (2018) [57] 12 months (article is 54 months follow-up)	Individual: Participate in disease self-management activities Healthcare Team: Pharmacists delivered disease self-management (telehealth) education every 2 weeks until BP control was achieved, then frequency was reduced; pharmacists communicated treatment goals, progress with primary care physicians, would request medication augmentation as necessary	Usual care	There were significant intervention effects on systolic BP for the first 18 months and diastolic BP for the first 12 months. At 54 months, there were no significant intervention effects on systolic (*p* = 0.18) or diastolic (*p* = 0.37) BP
Margolis (2022) [58] 12 months	Individual: Participate in disease self-management activities to achieve BP treatment goals; Participate in home BP telemonitoring Healthcare Team: Pharmacists delivered disease self-management (telehealth) education until BP control was achieved, then frequency was reduced; pharmacists communicated treatment goals, progress with primary care physicians, would request medication augmentation as necessary	Clinic-based care	There were significant decreases in systolic BP for both clinic-based care and telehealthcare, but there were no between-group differences in systolic BP change (*p* = 0.45)
McClintock (2017) [59] 12 weeks	Individual: Participate in individualized program activities to improve antihypertensive medication adherence, hypertension control; Interventionist-delivered pharmacotherapy for depression and hypertension management; engage in patient priority planning with interventionist (i.e., personalized management plan) Healthcare Team: Interventionists developed an intervention plan with participants that addressed both biomedical, social determinants of health needs (including developing community resource guide); interventionists shared patient priorities shared with clinical team	“Basic Intervention” (i.e., individualized program and depression-hypertension treatment integration without patient prioritized plan)	Participants in the enhanced intervention (basic and patient prioritized planning) had significantly improved systolic (*p* = 0.003) and diastolic (*p* = 0.019) BP compared to the basic intervention group
Nguyen-Huynh (2022) [60] 12 months (follow-up up to 48 months)	Individual: Participate in individualized lifestyle coaching sessions (Lifestyle Coaching condition); Participate in hypertension education activities (Enhanced Pharmacotherapy Monitoring condition) Healthcare Team: Enhanced Pharmacotherapy Monitoring condition: Research nurse coordinator and pharmacist team augmented medication treatment (e.g., optimize thiazine dosing, increase spironolactone prescribing for resistant hypertension); nurse-provided resource linking within healthcare network	Usual care	There were no significant effects of the enhanced pharmacotherapy intervention on BP control at 12, 24, and 48 months posttest. There were significant effects of the lifestyle coaching on BP control at 24 (*p* = 0.001) and 48 months (*p* = 0.006) posttest
Planas (2012) [61] 9 months	Individual: Participate in diabetes education and coaching on self-management skills Healthcare Team: Pharmacists completed comprehensive medication audit, recommended drug therapy changes with participant and conveyed to primary care physician	Usual care	There were significant intervention effects on systolic (*p* = 0.01) but not diastolic (*p* > 0.05) BP
Prezio (2013) [62] 12 months	Individual: Participate in 1-on-1 health coaching sessions with community health workers Healthcare Team: Community health workers facilitated physician contact, assisted participants with special visits, and provided disease self-monitoring materials (e.g., glucose monitors and testing strips)	Usual care	There were no significant intervention effects on systolic or diastolic BP
Rosas (2015) [63] 12 months (followed up to 24 months)	Individual: Individual and group-based diet and exercise intervention (case management and case management/community health worker condition); Participate in disease self-management activities as discussed with team Healthcare Team: Care coordination with case manager (and community health worker if assigned to that condition), physician; case management/community health worker arm only: inclusion of community health worker in care coordination, fostering family support, enhancing participant success in food negotiations, identifying	Usual care	There were no significant intervention effects for either case management or case management/community health worker teams in systolic or diastolic BP at 6, 12, or 24 months Among men, those in the case management/community health worker team intervention achieved a greater systolic and diastolic BP compared to case management at 24 months (*p* < 0.05)
Scott (2006) [64] 9 months	Individual: Participate in education appointments and disease self-management activities to achieve therapy goals developed with pharmacist Healthcare Team: Pharmacist-led diabetes education appointments, including referrals, implementing pharmacotherapy management recommendations approved by provider, and continuing goals/long-term plans	Usual care	At 9 months, there were significant intervention effects in systolic BP (*p* = 0.023). There were no significant intervention effects on diastolic BP
Vaughan (2017) [65] 6 months	Individual: Participate in comprehensive diabetes group visits and self-management activities Healthcare Team: Community health worker-led diabetes group visits and participant support between classes, including diet and medication adherence, reminders, and resource linking, as necessary	Usual care	There were no significant intervention effects on systolic (*p* = 0.89) or diastolic (*p* = 0.39) BP There were no significant intervention effects in the percent of participants who met target BP goals for either systolic (88% in intervention vs. 88% in control; *p* > 0.99) or diastolic (96% in intervention vs. 92% in control; *p* = 0.56) BP
Von Korff (2011) [66] 12 months	Individual: Participate in disease self-management and monitoring to achieve treatment goals Healthcare Team: Nurse, psychiatrist, primary care physician, and psychologist team meetings to review cases, patient progress; Nurse communicated drug treatment recommendations for primary care physician to enact; after achieving target levels, nurse and patient co-develop maintenance plan (and treatment intensification, if necessary)	Usual care	There were significant intervention effects on systolic BP at 6 and 12 months. At 12 months, the differences in systolic BP change between the intervention and control groups were significant (5.1 mmHg)
Zillich (2005) [67] 3 months	Individual: One-on-one disease education sessions with pharmacist; Participate in BP self-monitoring activities Healthcare Team: Pharmacist developed treatment recommendations based on participant self-monitoring data; physician and pharmacist co-develop treatment plan targeting both medication and lifestyle	Low-intensity intervention (BP assessment by pharmacist)	Systolic BP declined in both the low- and high-intensity intervention; there were no significant between-group differences (*p* = 0.12) There were significant between-group differences in diastolic BP favoring the high-intensity intervention (*p* = 0.03)
Individual and Healthcare Team and Health Policies			
Holtrop (2017) [68] 16 months	Individual: Participate in individualized behavioral changes as discussed with care manager Healthcare Team: Embedded care manager to provide patient resources (e.g., behavior change strategies, develop community resource guide) Health Policies: Care management enhancements to electronic health records (e.g., flagging gaps in chronic care, templates for disease management)	Usual care	There were no significant changes in systolic BP over time for either the intervention or control group
Huebschmann (2012) [69] 3 months	Individual: Participate in self-management and monitoring activities Healthcare Team: Outreach coordinator worked with patients to schedule BP-focused appointments with primary care physician; would facilitate future appointment scheduling if patient BP remained elevated Health Policies: Outreach coordinator-delivered electronic prompts to physicians prior to BP-related appointments with patients (including previous BP readings; JNC 7 guidelines and treatment)	Usual care	While there were changes in clinical inertia, there were no intervention effects on systolic (*p* = 0.50) or diastolic (*p* = 0.71) BP
Murray (2004) [70] 6 months	Individual: Receive medication counseling from pharmacists; participate in individualized treatment activities (e.g., exercise, smoking cessation) Healthcare Team: Pharmacists use computer systems to determine medication regime; serves as liaison to communicate medication changes between the participant and physician Health Policies: Computer-based treatment ordering system for physicians and/or pharmacists	Usual care (factorial design; physician and pharmacist intervention as separate components)	There were no statistical or clinically significant intervention effects on systolic or diastolic BP
Piatt (2010) [71] 12 months (up to 3 years follow-up)	Individual: Participate in diabetes self-management education and support group (CCM intervention only) Healthcare Team: Physician diabetes education lecture on problem-based learning (Both PROV, CCM interventions); On-site certified diabetes educator for patient and/or provider consultation (CCM intervention only) Health Policies: Providers encouraged to redesign processes for seeing patients with diabetes for routine visits (CCM intervention only); Chart audit completed by certified diabetes educator (Both PROV, CCM interventions)	Usual care (chart audit report and decision support)	At 12 months, there were significant effects of both the chronic care model and provider education-only interventions on systolic BP. These results were not sustained at 36 months
Ralston (2009) [72] 12 months	Individual: Participate in disease self-management activities after developing action plan with care manager Healthcare Team: Web-based case manager provided disease self-management education; collaborated on decision support with patient and provider; integrated patient communications in ongoing care (communicate needs with provider) Health Policies: Established provider decision support using participant self-monitoring inputs (e.g., glucose reading)	Usual care	There were no significant intervention effects on systolic (*p* = 0.93) or diastolic (*p* = 0.91) BP
Rothman (2005) [73] 12 months	Individual: Disease education and counseling, medication management training from pharmacists Healthcare Team: Diabetes care coordinator available for patients to discuss barriers to care, health education, insurance problems, or low health literacy; Pharmacists available for one-on-one or joint appointments with the patient and physician Health Policies: Evidence-based treatment algorithms applied to improve medication use	One-time management session from pharmacist and usual care	The intervention group had significantly greater improvement in systolic BP compared to the control group (difference of 9 mmHg)
Roumie (2006) [74] 6 months	Individual: Educational information on lifestyle modifications and medication adherence for improving BP control. The letter also linked to the American Heart Association for more details, e.g., disease self-management information Healthcare Team: Physician educational email on the JCN 7 guidelines for treating hypertension Health Policies: Electronic notification sent to the physician by the pharmacy including dates and values of patient’s last 3 BP measures and offered treatment options	Factorial design comparing provider education alone, provider education and alert, and provider education and alert and patient education	Those who received the provider education and alert and patient education intervention had better BP control; providers of those in this intervention also reported more patients with a systolic BP of 140 mmHg or less. There were no significant intervention effects on diastolic BP
Schoenthaler (2020) [75] 6 months	Individual: Participate in health coaching sessions with medical assistants to improve medication adherence Healthcare Team: Physician-medical assistant “teamlets” collaborate on patient care Health Policies: Office system support: use EHR to identify Latino patients with uncontrolled hypertension to refer them to bilingual medical assistant	Usual care	There were significant improvements in systolic and diastolic BP for both groups; there were no significant intervention effects for either outcome
Svarstad (2013) [76] 6 months (up to 12 month outcomes)	Individual: Take-home toolkits including pedometer, medication boxes, BP self-management information packets; received self-report tools from pharmacists Healthcare Team: Team-based training for pharmacists, pharmacy technicians on how to use clinical tools to address patient barriers and improve BP control Health Policies: Clinical toolkits that included algorithms to address patient barriers, provide physician feedback; Developing an on-site BP clinic (including providing furniture and equipment for a semiprivate BP station) for pharmacists to deliver counseling, tailored intervention, and patient tool kit	Information pamphlet on BP control for patients; letter with JNC-7 guidelines to physicians and pharmacists	At 6 months, intervention participants had significantly better systolic BP changes (−12.62 vs. −5.31 mm Hg, *p* < 0.001), greater BP control (50% vs. 36%, *p* = 0.01), and greater medication refill adherence (60% vs. 34%, *p* < 0.001). At 12 months (6 months after discontinuation), refill adherence (*p* < 0.001) and systolic BP (*p* = 0.004) were still better in the intervention group, but BP control was not different (*p* > 0.05)
Svetkey (2009) [77] 18 months	Individual: Group-based diet, physical activity, and medication adherence intervention Healthcare Team: Physician training modules on JNC-7 guidelines (counted toward CME credits); Community health advisors offered lifestyle counseling for participants during, after group-based intervention Health Policies: Treatment algorithm and decision tree based on JNC-7 guidelines; physicians completed quality improvement forms that were converted to personalized quarterly feedback forms	MD-Control: Usual care for physician Patient-Control: One-time interventionist visit to receive advice, written materials	At 6 months, there was a significant effect of the physician and patient intervention on systolic BP (*p* = 0.03; decrease of 9.7 mmHg). Differences between the groups did not persist at 18 months
Talavera (2021) [78] 6 months	Individual: Participate in group-health education classes led by community health workers; Participate in disease self-management activities to achieve treatment goals Healthcare Team: Shared treatment plan between physician and specialty behavioral health provider and care coordination to facilitate treatment; up to 4 integrated medical visits with physician, specialty behavioral health provider; collaboration between physician, patient, and specialty behavioral health provider on treatment goals Health Policies: Co-location of clinical team, warm hand-off from medical to specialty behavioral health provider	Usual care	There were no significant intervention effects on systolic (*p* = 0.86) or diastolic (*p* = 0.09) BP
Towfighi (2021) [79] 12 months	Individual: Participate in disease self-management and monitoring activities to achieve health goals Healthcare Team: Community health workers addressed medication adherence; provided education and self-management training; offered resources/referrals to participants to address social determinants of health; served as a liaison between patients, healthcare system; conducted at least 3 home visits; participants offered at least 3 advanced practice clinician meetings, where medication was prescribed/titrated Health Policies: Electronic decision support for clinicians; protocol-driven risk factor management	Usual care	There were no significant intervention effects for systolic BP (*p* = 0.46). When restricted to those with baseline systolic BP ≤ 130 mmHg, a similar nonsignificant result was found (*p* = 0.57)
Vinicor (1987) [80] 2 months (followed up to 26 months)	Individual: Participate in disease self-management education training Healthcare Team: Physician education on common diabetes problems and management/diagnostic strategies for handling problems; following physician seminars, “Consultation Conference” small group discussions with faculty and residents to discuss protocol implementation issues Health Policies: Protocol-based physician reminders	Usual care (factorial design comparing usual care, patient education, physician education, or patient and physician education)	There were no intervention effects on systolic BP. There were significant effects of the patient and physician education intervention on diastolic BP (effect size = 0.47)
Wang (2012) [81] 18 months	Individual: Participate in disease self-monitoring activities; those maintaining adequate BP control did not activate intervention Healthcare Team: If home BP control was inadequate, nurse contacted patients to administer tailored modules to improve self-management; medication management condition: Nurse notified physician when medication treatment needed to be augmented Health Policies: Medication management condition: Nurses provided standardized, evidence-based decision support tool for hypertension medication management	Usual care (4 conditions: usual care, behavioral management alone, medication management alone, or behavioral and medication management)	Participants who received either behavior management alone or medication management alone showed significant gains in BP control at 12 months, but these effects were not sustained at 18 months. There was no significant effect of combined management on BP control
Welch (2011) [82] 12 months	Individual: Participate in diabetes education course and self-management activities Healthcare Team: Nurse who leads diabetes education coordinates diabetes medication treatment with primary care physician Health Policies: Patient risk profiles developed from participant’s lab results, including summary of diabetes self-management behaviors and barriers for physicians to discuss with participant	Educational control	There were no significant intervention effects on systolic (*p* = 0.11) or diastolic (*p* = 0.17) BP. There were also no significant intervention effects on the percentage of patients with BP < 130/80 mmHg (*p* = 0.09)
Individual and Community Environment			
Ard (2017) [83] 6 months	Individual: Group weight loss intervention targeting physical activity and diet Community Environment: County-level grants that key stakeholders could apply to support local initiatives (e.g., community garden, enhance walking trail)	Weight loss only intervention	Both groups had significant reductions in systolic BP (weight loss only: 1.9 mmHg; weight loss plus: 4.0 mmHg) and diastolic BP: (weight loss only: 1.7 mmHg; weight loss plus: 2.9 mmHg). No between-group differences
Brewer (2022) [84] 6 months	Individual: Psychoeducation on hypertension management; participation in diet or physical activity “lifestyle journey” Community Environment: Between-church competition/social incentives; church-level self-monitoring posted in public areas	Delayed intervention	No significant intervention effects on systolic BP (*p* = 0.14) or diastolic BP (*p* = 0.37), but the intervention significantly improved the overall Life’s Simple 7 score
Brown (2015) [85] 12 months	Individual: Self-help materials for BP Community Environment: Environmental and social changes encouraged in the parish, including food provided at parish functions and providing church programs to encourage healthy cooking, physical activity	Active control (received skin cancer awareness materials)	No significant intervention effect for systolic BP (*p* = 0.86) or diastolic BP (*p* = 0.16)
Derose (2019) [86] 5 months	Individual: Participate in physical activity, nutrition, education classes Community Environment: Community garden grown on church grounds, maintained by church members; inventory of local food and exercise establishments identified; church members brainstorm points for advocacy	Waitlist control	There were no significant intervention effects on systolic (*p* = 0.80) or diastolic (*p* = 0.75) BP
Kerr (2018) [87] 12 months	Individual: Developed goals with peer leader in 1-on-1 sessions; Participated in individual counseling and physical activity self-monitoring, group education sessions, and group walking sessions Community Environment: Peer leaders advocated for local environment improvements (e.g., walk audits, received training on engaging with policy makers and city officials)	Healthy aging control (attention and social control who received 9 group education sessions and 4 general health calls)	There were significant intervention effects for systolic (*p* < 0.01) and diastolic (*p* < 0.05) BP at 6 months, but this was not sustained at 12 months
Nafziger (2001) [88] 5 years	Individual: Participate in wellness activities, including education, lifestyle activities, and health screening events Community Environment: Local health committees developed in 24 communities to develop health promotion programs tailored to community’s needs; Community leaders formed “Healthy Heart Advisory Committees” to provide general guidance; Engaging local media outlets to deliver education materials; Development of local walking groups; Workplace and neighborhood risk factor screening	Usual care	Systolic BP was significantly improved among the intervention group, but there were no intervention effects for diastolic BP
Paskett (2018) [89] 12 months	Individual: Walking by Faith: Participate in diet intervention activities to meet individualized health goals Ribbons of Faith: Participate in education sessions; church members encouraged to complete cancer screenings Community Environment: Walking by Faith: Community navigators, project staff co-developed strategies to support environmental approaches to increase physical activity, including setting up walking courses, group walks, walking challenges; identified safe walking paths; used donations from local businesses and organizations to encourage exercise Ribbons of Faith: Health fairs; cancer education inserts shared in church bulletins	Compared two interventions (Ribbons of Faith vs. Walking by Faith)	There were no significant effects in systolic BP for either the Walk by Faith or Ribbons of Faith intervention
Seguin (2018) [90] 6 months	Individual: Skills-based classes on nutrition and physical activity Community Environment: HEART Club civic engagement, including development of community guides with resources for health, healthcare and wellness, and community change; HEART Clubs identified environmental issues relevant to their community, followed system to article and evaluate action steps to advocate for community change	Education-only control	There were no significant intervention effects on systolic (*p* = 0.37) or diastolic (*p* = 0.37) BP
Seguin-Fowler (2020) [91] 6 months	Individual: Skills-based classes on nutrition and physical activity; group discussions on building social support for exercising, improving diet outside of fitness courses Community Environment: Increased civic engagement, including grocery store tours and town walking audits; HEART clubs identified environmental issues relevant to their community, followed system to article and evaluate action steps to advocate for community change	Delayed intervention control	There were no significant intervention effects on systolic or diastolic BP, but there were significant intervention effects on the Life’s Simple 7 total score (*p* < 0.001). The intervention effect on Life’s Simple 7 was not present when restricted to adults > 60 years old
Individual and Family/Social Support			
Baranowski (1990) [92] 14 weeks	Individual: Individual fitness goals during exercise component Family/Social Support: Family-level behavioral counseling, education (separate for children and parents), aerobic activity, and snacks	No-contact control	No significant changes in BP for parents or children except in fourth-phase diastolic BP among children favoring the intervention group
Matthan (2022) [93] 12 months	Individual: Group-based physical activity for children Family/Social Support: Families received education materials on diet, exercise; families participated in skill-building group activities (e.g., cooking); Parents-only education/support sessions	Usual care	There were no significant intervention effects for either systolic (*p* = 0.779) or diastolic (*p* = 0.786) BP. There were no changes in BP over time for either treatment condition
Rosland (2022) [94] 12 months (followed up to 15 months)	Individual: Participate in disease self-management coaching session with care partner; one-on-one health coaching sessions to discuss progress on achieving health goals Family/Social Support: One-on-one health coaching sessions to discuss how to support participant’s disease self-management activities; participate in preparation calls before scheduled clinic visits with participant, dyad coach	Enhanced usual care (disease self-management materials)	There were no significant intervention effects on systolic BP (*p* = 0.18)
Trief (2016) [95]; Trief (2019) [96] 4 months (followed up to 12 months)	Individual: Participate in comprehensive diabetes education (all training arms); participate in disease self-management intervention (individual calls) Family/Social Support: Participate in couples’ self-management intervention (couples calls)	Usual care (diabetes self-management education)	There were no significant intervention effects on systolic or diastolic BP for participants with type 2 diabetes There were no significant intervention effects on systolic BP for care partners of those with type 2 diabetes. There were significant intervention effects on diastolic BP at 4, 8, and 12 months (couples counseling > diabetes education; couples counseling > individual counseling at 12 months only). There were also significant effects of the individual counseling on their care partner’s diastolic BP at 4 months (compared to diabetes education), but this was not sustained
Wieland (2018) [97] 12 months	Individual: Participate in individual health goal setting with family health promoter; engage in activities to meet health goals Family/Social Support: Participate in family health goal setting with family health promoter, home visits, and education activities	Delayed intervention	There were no significant intervention effects on systolic or diastolic BP at 6 (*p* = 0.11 and 0.41, respectively) or 12 months (*p* = 0.13 and 0.48, respectively)
Yates (2015) [98] 6 months (30-year trajectories modeled)	Individual: Participate in individual counseling, education, and goal-setting activities (spouse/partner); participate in individual counseling, education, and goal-setting activities (participant who underwent coronary artery bypass Family/Social Support: Participate in group fitness activities with participant and spouse/partner	Usual care (education and counseling advice)	There was a marginally significant intervention effect on 30-year estimated systolic BP on the spouse/partner (partial eta-squared = 0.002, *p* = 0.06)
Individual and Family/Social Support and Healthcare Team			
Haskell (2006) [99] 12 months	Individual: Participate in disease self-management activities Family/Social Support: Family activated by disease management team to support participants risk-reduction efforts Healthcare Team: Physician, nurse/nurse practitioner, and dietitian co-developed treatment plan (including lifestyle and medical management)	Usual care	There were significant posttest differences in systolic and diastolic BP favoring the intervention group (*p* < 0.01)
Levine (2003) [100] 40 months	Individual: Participate in community high BP education and educational pamphlet; Participate in lifestyle/behavioral activities to meet treatment goals discussed with community health worker Family/Social Support: Family or friends of participant taught how to provide functional, social support by community health worker Healthcare Team: Conduct home visits with participants to provide education, lifestyle support, resource linking, and access to care, health insurance, or other system-related factors	Psychoeducational control (education and pamphlet only)	There were no significant intervention effects on systolic or diastolic BP
Pearce (2008) [101] 12 months	Individual: Participate in individualized patient education session with support person; patient-specific educational newsletters on cardiovascular risk factor control Family/Social Support: Participate in individualized patient education session; support person-specific educational newsletters on facilitating cardiovascular risk factor control Healthcare Team: Registered nurse patient educator delivered education sessions and newsletters	Individualized patient education without inclusion of support person education	There were no significant intervention effects on systolic BP
Pérez-Escamilla (2015) [102] 12 months (followed up to 18 months)	Individual: Participate in tailored intervention activities to achieve treatment goals (e.g., self-monitoring, medication adherence, nutrition) Family/Social Support: Families allowed to participate during home sessions with participant, community health worker if endorsed by participant Healthcare Team: Community health worker and participant co-developed type 2 diabetes self-management plan; community health worker participated in weekly meetings with primary care physicians’ medical teams and dieticians to discuss treatment options, participant concerns or barriers	Usual care	There were no significant intervention effects on systolic BP across 3, 6, 12, or 18 months (*p* range: 0.279–0.313.)
Healthcare Team and Health Policies			
Petersen (2013) [103] 20 months	Healthcare Team: Physicians received financial incentives for achieving BP control, appropriately responding to uncontrolled BP, and/or prescribing antihypertensive medications; physician financial incentives provided from directors of participating regional hospitals (contributed USD 250,000) Health Policies: Practice-level incentives for achieving BP control, appropriately responding to uncontrolled BP, and/or prescribing antihypertensive medications (regardless of randomization to individual component)	Usual care (Factorial design comparing individual-level incentives, practice-level incentives, and combination vs. usual care)	Individual but not practice-level or combined incentives resulted in greater BP control. This effect was not sustained after a washout period of 18 months
Peterson (2008) [104] 12 months (up to 24 months follow-up)	Healthcare Team: Practice redesign including establishment of local physician champion who facilitated previsit planning, physician reminders Health Policies: Electronic diabetes registry of high-risk participants to track operational activity, clinical measures; reports reviewed in monthly team meetings	Usual care	The percentage of patients who met the target BP levels was significantly higher among the intervention clinics (*p* = 0.05)
Weber (2010) [105] 9 months	Healthcare Team: Pharmacist–physician team develop medication regimen for patient, incorporating clinical pharmacist’s patient interview Health Policies: Clinical pharmacists reviewed patient data, interviewed patients to identify current treatment strategies, potential barriers to achieving BP goals (met at least 1x/every 2 months)	Usual care	Ambulatory systolic BP was reduced to a greater extent in the intervention compared to control group (daytime systolic BP, nighttime systolic BP, 24-h systolic BP; *p* < 0.001). More participants in the intervention had controlled BP at the study end compared to controls (24-h BP; *p* < 0.001)
Individual and Healthcare Team and Community Environment			
Victor (2011) [106]; Rader (2013) [107] subgroup analysis 10 months	Individual: Attend healthcare and barber appointments (receive a free haircut for every high BP referral card signed by physician and returned to barber) Healthcare Team: Customers who did not have physicians were connected with local community physicians and safety-net clinics Community Environment: BP checks offered during haircuts at local barbershop; personalized peer-based health messaging incorporated through conversations, posters, and barbers modeling desired treatment-seeking behavior; barbers received incentives per customer’s recorded BP, referral, and BP cards returned by patrons with physician’s signature (documenting physician–customer contact)	AHA-provided pamphlets (High Blood Pressure in African Americans) made available to control barbershops	There was no significant effect of the intervention on diastolic BP, but there was a marginal intervention effect for systolic BP change (*p* = 0.08) When barbers referred customers to hypertension specialists, there was a systolic BP reduction of 21 mmHg (*p* < 0.0001) compared to the control. When referred to primary care physicians, there were no significant differences compared to the control group (4 mmHg; *p* = 0.31)
Victor (2018) [108]; Victor (2019) [109] 6 months (followed up to 12 months)	Individual: Participants invited to share health stories with pharmacist to display on barbershop walls Healthcare Team: Pharmacists collaborated with physician hypertension specialists on treatment regimen Community Environment: Pharmacists stationed in barbershops to prescribe medication, assess BP and plasma electrolyte levels, and encourage lifestyle changes; barbers encouraged participants to follow-up with pharmacists, trained on BP assessment	Educational materials and BP screener	At 6 months, the mean reduction in systolic BP was 21.6 mmHg greater in the intervention compared to control group (*p* < 0.001). The mean reduction in diastolic BP was 14.9 mmHg greater in the intervention compared to control group (*p* < 0.001). A BP level of less than 130/80 mmHg was achieved among 63.6% of the intervention vs. 11.7% of the control (*p* < 0.001) At 12 months, the mean reduction in systolic BP was 20.8 mmHg greater in the intervention compared to control group (*p* < 0.0001). The mean reduction in diastolic BP was 14.5 mmHg greater in the intervention compared to control group (*p* < 0.0001). A BP level of less than 130/80 mmHg was achieved among 68% of the intervention vs. 11% of the control (*p* < 0.02)
Individual and Community Environment and Health Policies			
Pereira (2020) [110] 12 months	Individual: Education, behavioral cues, goal-setting, and relapse prevention provided through newsletters, 1-on-1 coaching sessions (both STAND+, MOVE+ conditions) Community Environment: Desk-based ergonomic setup at work (e.g., standing desks) (STAND+ condition only) Health Policies: Establishment of workplace health leaders and advocates to provide links between worksite employees, management, and study team (both STAND+, MOVE+ conditions); Worksite leaders and advocates share new intervention initiatives with employees (both STAND+, MOVE+ conditions)	Comparison of two interventions (MOVE+ vs. STAND+)	There were no significant between-group differences in either systolic or diastolic BP When restricting the analysis to those with dysgylcemia, the effect of the STAND+ on systolic BP was stronger, albeit nonsignificant (difference [95% CI] −6.6 [−15, 1.7])
Wilcox (2013) [111] 15 months	Individual: Church leadership and kitchen staff participate in didactic training on physical activity, healthy eating, and adopting meals to meet DASH recommendations; Church leaders monitor healthful behaviors (e.g., wear pedometer, disseminate health information from pulpit) Community Environment: Churches provide physical activity programs at church; offer physical activity before or during services; provide exercise equipment at churches; provide skills-based classes; church leadership negotiate for discounted YMCA membership; lobby for community centers; Establish a physical activity ambassador who incorporates physical activity into church functions; Use competitions between churches to increase enjoyment, incentivize activities; Have pastors lead efforts in growing gardens, attend farmer’s market Health Policies: Establish a church health commission; Pastor reports to include physical activity, healthy eating activities for accountability; Update church policies for food and drink at all events; provide certificates for cooks who attend training and adhere to guidelines; churches received up to USD 1000 to assist with implementing health action plan	Delayed intervention	There were no significant intervention effects on systolic (*p* = 0.58) or diastolic (*p* = 0.91) BP
Individual and Health Policies			
Skolarus (2023) [112] 12 months	Individual: Receive tailored healthy behavior text messages, including recommendations on where to find discounted medications; prompted self-managed BP monitoring with personalized feedback Health Policies: Facilitated scheduling, transportation to primary care appointments	Passive monthly text reminders (factorial design comparing 3 mHealth strategies)	Systolic BP declined similarly across all groups; there were no significant differences by intervention group
Individual and Family/Social Support and Healthcare Team and Community Environment			
Campbell (2014) [113] 5 years	Individual: Child attended full-day childcare 5 days/week, 50 weeks/year, for 5 years, starting at 2 months old Family/Social Support: Parents received social service support as requested Healthcare Team: Medical team conducted physical exams and counseled parents on child health, nutrition, growth, and development Community Environment: Childcare center provided free primary pediatric care, nutrition, and referrals as appropriate	Usual care	At age 30, men from the intervention group had significantly lower systolic BP (*p* = 0.018) and diastolic BP (*p* = 0.024). There were no significant effects for women.

Note. BP = Blood pressure.

## Data Availability

Data sharing is not applicable to this article as no datasets were generated or analyzed during the current study. An Open Science Framework protocol was created and can be found here https://osf.io/hzsr6/?view_only=30b08c6976774136aefd04f70f14f8f0 (accessed on 1 February 2024).

## Appendix A

### Appendix A.1. Search Terms by Database

**Table:**

**Database**	**Search Terms**
Cochrane Library	“Hypertension” OR “blood pressure” AND “intervention” OR “trial” AND “adult” Additional filters: Content type = Cochrane reviews; trials No date restriction “Search Word Variations” selected Cochrane Group = Hypertension Note: This database was searched to identify whether a similar review has been undertaken. Relevant reviews will be used to conduct hand searches for additional articles, if available.
EMBASE	(‘hypertension’/exp OR hypertension OR ‘blood pressure’) AND (‘hairdresser’ OR ‘diet therapy’ OR exercise OR ‘health coaching’ OR ‘public health’ OR ‘prevention and control’ OR ‘lifestyle’) AND english:la AND adult AND ([controlled clinical trial]/lim OR [randomized controlled trial]/lim) AND ([adult]/lim OR [aged]/lim OR [middle aged]/lim OR [very elderly]/lim OR [young adult]/lim) AND ‘article’/it
PsycINFO	MA “hypertension” OR TX (blood pressure or hypertension or hypertension or htn or elevated blood pressure) AND TX multilevel intervention OR TX lifestyle intervention OR TX health coach OR MA barber* OR TX (diet or nutrition or food habit or eating habit or lifestyle or food) OR TX (exercise or physical fitness or physical activity or exercise therapy) OR TX community health Search Limiters: Peer reviewed, publication type: peer reviewed journal; English language; Language: English; Age Groups: Adulthood (18 yrs & older), Young Adulthood (18–29 Years), Thirties (30–39 yrs), Middle Age (40–64 yrs), Aged (65 yrs & older), Very Old (85 yrs & older); Population Group: Human; Methodology: CLINICAL TRIAL; Exclude dissertations Search Expanders: Apply related words; also search within full text of the articles; apply equivalent subjects Search Modes: Boolean/phrase
PubMed	((adult[Filter] OR middleagedaged[Filter])) AND ((((Hypertension[MH] OR Blood Pressure[MH] or Cardiovascular Health[MH]) OR (“Life’s Simple 7”[tw] or “Life’s Simple Seven”[tw] OR “Essential 8”[tw] OR “systolic”[tw] or “diastolic”[tw])) AND (Antihypertensive Agents/therapeutic use*[mh] OR Barbering*[MH] OR Cardiovascular Diseases/prevention & control [MH] OR Preventive Health Services [MH] OR Health Promotion/methods* [MH] OR “barber*”[tw] OR “salon”[tw] OR “health coach*”[tw] OR “Exercis*”[tw] OR “diet*”[tw] OR “lifestyle”[tw] OR “life style”[tw] OR “life-style”[tw] OR “nontraditional health care”[tw] or “multilevel”[tw] OR “multi-level”[tw] OR “communit*”[tw] AND clinical trial[publication type] or random*[title/abstract])) AND (“intervention”)) Note: * indicates wildcard term

### Appendix A.2. PRISMA Checklist

**Table:**

**Section and Topic**	**Item #**	**Checklist Item**	**Location Where Item Is Reported**
TITLE			
Title	1	Identify the report as a systematic review.	1
ABSTRACT			
Abstract	2	See the PRISMA 2020 for Abstracts checklist.	2
INTRODUCTION			
Rationale	3	Describe the rationale for the review in the context of existing knowledge.	5
Objectives	4	Provide an explicit statement of the objective(s) or question(s) the review addresses.	5
METHODS			
Eligibility criteria	5	Specify the inclusion and exclusion criteria for the review and how studies were grouped for the syntheses.	6–7
Information sources	6	Specify all databases, registers, websites, organizations, reference lists and other sources searched or consulted to identify studies. Specify the date when each source was last searched or consulted.	6
Search strategy	7	Present the full search strategies for all databases, registers and websites, including any filters and limits used.	Appendix A.1 (p. 26)
Selection process	8	Specify the methods used to decide whether a study met the inclusion criteria of the review, including how many reviewers screened each record and each report retrieved, whether they worked independently, and if applicable, details of automation tools used in the process.	6–8
Data collection process	9	Specify the methods used to collect data from reports, including how many reviewers collected data from each report, whether they worked independently, any processes for obtaining or confirming data from study investigators, and if applicable, details of automation tools used in the process.	7–9
Data items	10a	List and define all outcomes for which data were sought. Specify whether all results that were compatible with each outcome domain in each study were sought (e.g., for all measures, timepoints, analyses), and if not, the methods used to decide which results to collect.	7–8
	10b	List and define all other variables for which data were sought (e.g., participant and intervention characteristics, funding sources). Describe any assumptions made about any missing or unclear information.	8
Study risk of bias assessment	11	Specify the methods used to assess risk of bias in the included studies, including details of the tool(s) used, how many reviewers assessed each study and whether they worked independently, and if applicable, details of automation tools used in the process.	8
Effect measures	12	Specify for each outcome the effect measure(s) (e.g., risk ratio, mean difference) used in the synthesis or presentation of results.	8
Synthesis methods	13a	Describe the processes used to decide which studies were eligible for each synthesis (e.g., tabulating the study intervention characteristics and comparing against the planned groups for each synthesis (item #5)).	10–11
	13b	Describe any methods required to prepare the data for presentation or synthesis, such as handling of missing summary statistics, or data conversions.	8
	13c	Describe any methods used to tabulate or visually display results of individual studies and syntheses.	8
	13d	Describe any methods used to synthesize results and provide a rationale for the choice(s). If meta-analysis was performed, describe the model(s), method(s) to identify the presence and extent of statistical heterogeneity, and software package(s) used.	9
	13e	Describe any methods used to explore possible causes of heterogeneity among study results (e.g., subgroup analysis, meta-regression).	9
	13f	Describe any sensitivity analyses conducted to assess robustness of the synthesized results.	NA
Reporting bias assessment	14	Describe any methods used to assess risk of bias due to missing results in a synthesis (arising from reporting biases).	8
Certainty assessment	15	Describe any methods used to assess certainty (or confidence) in the body of evidence for an outcome.	NA
RESULTS			
Study selection	16a	Describe the results of the search and selection process, from the number of records identified in the search to the number of studies included in the review, ideally using a flow diagram.	27 (Figure 1)
	16b	Cite studies that might appear to meet the inclusion criteria, but which were excluded, and explain why they were excluded.	7
Study characteristics	17	Cite each included study and present its characteristics.	9–11
Risk of bias in studies	18	Present assessments of risk of bias for each included study.	29–41 (Table 2)
Results of individual studies	19	For all outcomes, present, for each study: (a) summary statistics for each group (where appropriate) and (b) an effect estimate and its precision (e.g., confidence/credible interval), ideally using structured tables or plots.	42–65 (Table 3)
Results of syntheses	20a	For each synthesis, briefly summarize the characteristics and risk of bias among contributing studies.	9–14
	20b	Present results of all statistical syntheses conducted. If meta-analysis was performed, present for each the summary estimate and its precision (e.g., confidence/credible interval) and measures of statistical heterogeneity. If comparing groups, describe the direction of the effect.	NA
	20c	Present results of all investigations of possible causes of heterogeneity among study results.	NA
	20d	Present results of all sensitivity analyses conducted to assess the robustness of the synthesized results.	NA
Reporting biases	21	Present assessments of risk of bias due to missing results (arising from reporting biases) for each synthesis assessed.	NA
Certainty of evidence	22	Present assessments of certainty (or confidence) in the body of evidence for each outcome assessed.	NA
DISCUSSION			
Discussion	23a	Provide a general interpretation of the results in the context of other evidence.	15–18
	23b	Discuss any limitations of the evidence included in the review.	16–17
	23c	Discuss any limitations of the review processes used.	17
	23d	Discuss implications of the results for practice, policy, and future research.	18
OTHER INFORMATION			
Registration and protocol	24a	Provide registration information for the review, including register name and registration number, or state that the review was not registered.	6
	24b	Indicate where the review protocol can be accessed, or state that a protocol was not prepared.	6
	24c	Describe and explain any amendments to information provided at registration or in the protocol.	6 (OSF preregistration link)
Support	25	Describe sources of financial or non-financial support for the review, and the role of the funders or sponsors in the review.	2
Competing interests	26	Declare any competing interests of review authors.	1
Availability of data, code and other materials	27	Report which of the following are publicly available and where they can be found: template data collection forms; data extracted from included studies; data used for all analyses; analytic code; any other materials used in the review.	2, 6, 8

From: [124]. This work is licensed under CC BY 4.0. To view a copy of this license, visit https://creativecommons.org/licenses/by/4.0/ (accessed on 1 February 2024).

### Appendix A.3. Summary of Multilevel Interventions with Notable BP Outcomes and Population-Specific Effects

**Table:**

**Study (First Author, Year)**	**Intervention Levels**	**Population Highlights**	**BP Outcome**	**Sustained Effects?**	**Notable Moderators/Subgroups**
Ard, 2017 [83]	Individual and Community Environment	Black women aged 30–70	↓ SBP and DBP in both groups (significant within-group reductions)	Not reported	Not reported
Boulware, 2020 [27]	Individual and Healthcare Team	Black adults with uncontrolled hypertension	↓ SBP among all groups (no intervention effect)	No (not sustained beyond intervention)	Not reported
Crist, 2022 [32]	Individual and Healthcare Team and Health Policies	Adults aged 50+ who walk without assistance; senior/community centers serving low-income populations	↓ SBP (significant at 18 months); ↑ cost-effectiveness	No (not sustained at 24 months)	Not reported
Katon, 2010, 2012 [51,52]	Individual and Healthcare Team	Participants with diagnosis of diabetes and/or coronary heart disease	↓ SBP (significant)	No (Not sustained at 18- or 24-month follow-up)	Not reported
Petersen, 2013 [103]	Healthcare Team and Health Policies	Physicians who see adult patients in hospital-based settings	↑ BP control (via provider incentives)	Mixed (effect of individual-level incentives not sustained after 18-month washout)	Not reported
Svarstad, 2013 [76]	Individual and Healthcare Team and Health Policies	Black adults, 576 participants across 28 chain pharmacies	↓ SBP (significant); ↑ BP control; ↑ adherence	Partial (SBP and adherence sustained at 12 months, but not BP control)	Not reported
Victor, 2018, 2019 [108,109]	Individual and Healthcare Team and Community	Black men, barbershop-based	↓ SBP and DBP (significant)	Yes (12-month maintenance)	Greater effect among men with SBP ≥ 140 mmHg referred to hypertension specialists

Note: SBP = systolic blood pressure; ↑ = increased/higher value; ↓ = decreased/lower value. This table highlights illustrative examples of interventions from the review that demonstrated statistically significant reductions in systolic and/or diastolic blood pressure, sustained effects over time, and/or subgroup differences in effectiveness. It is not an exhaustive list but intended to support interpretability by summarizing promising intervention characteristics and contexts.
